# Implementation science frameworks and strategies to promote adoption of and adherence to oncology clinical practice guidelines in low- and middle-income countries: a scoping review

**DOI:** 10.1186/s44263-026-00297-4

**Published:** 2026-07-16

**Authors:** Anya Romanoff, Kathleen Lynch, Lily Martin, Olayide Agodirin, Shilpa Murthy, Olalekan Olasehinde, Chukwuma Okereke, Julia M. Jackman, Betel Yibrehu, William E. Rosa, Catherine Zivanov, Erica Mann, Joy O. Ogunmuyiwa, Colleen E. Witty, Victoria L. Mango, Olusegun Isaac Alatise, T. Peter Kingham, Rachel Vreeman, Benjamin O. Anderson, Jamie S. Ostroff

**Affiliations:** 1 Global Cancer Research and Training, Memorial Sloan Kettering Cancer Center, New York, NY USA; 2 Department of Global Health and Health System Design, Icahn School of Medicine Mount Sinai, New York, NY USA; 3 Department of Psychiatry and Behavioral Science, Memorial Sloan Kettering Cancer Center, New York, NY USA; 4 Department of Social and Behavioral Science, New York University School of Global Public Health, New York, USA; 5 New York University Health Sciences Library, New York University Grossman School of Medicine, New York University, New York, NY USA; 6 Department of Surgery, University of Ilorin Teaching Hospital, Ilorin, Nigeria; 7 Department of Surgery, Yale University, New Haven, CT USA; 8 Department of Surgery, Obafemi Awolowo University Teaching Hospitals Complex, Ile Ife, Nigeria; 9 Department of Surgery, Federal Medical Center Owo, Owo, Nigeria; 10 Department of Surgery, Washington University School of Medicine, St. Louis, MO USA; 11 Department of Surgery, Lankenau Medical Center, Wynnewood, Pennsylvania USA; 12 Department of Surgery and Global Health, University of Washington School of Medicine, Seattle, WA USA

**Keywords:** Oncology clinical practice guidelines, Implementation science, Frameworks, Strategies, Low- and middle-income countries, Scoping review

## Abstract

**Background:**

Clinical practice guidelines (CPGs) were developed to standardize and optimize cancer care delivery in low- and middle-income countries (LMICs). The aim of this scoping review is to identify implementation science (IS) frameworks and strategies to promote CPG adoption and adherence in LMICs.

**Methods:**

We identified studies that describe, develop, reference, or utilize IS frameworks or strategies to deliver or evaluate adoption of oncology CPGs in LMICs. Using the Preferred Reporting Items for Systematic Reviews and Meta-Analyses extension for Scoping Reviews (PRISMA-ScR), we searched Medline, Embase, Cochrane Central Register of Controlled Trials (CENTRAL), African Journals Online, Latin American and Caribbean Health Sciences Literature, Scopus, Web of Science, and PsycINFO on 3/21/2022, 12/20/2022, 01/26/2024, and 10/03/2025. Publications in all languages and of all study types were eligible for inclusion. Titles, abstracts, and full text were screened by two reviewers with conflicts resolved by a third reviewer. Excluded studies did not use an IS framework or strategy, did not focus on cancer care, or were conducted in a high-income country.

**Results:**

Searches identified 17,806 unique publications for title/abstract screening. 182 studies met criteria for full-text review; 35 were included. Twenty-four studies were country-specific, most commonly India (n = 5) and Nigeria (n = 5). The most frequent CPGs referenced were national/country-specific guidelines (n = 13) and Breast Health Global Initiative (BHGI) guidelines (n = 9). Only 16 full-text publications described original research to promote or evaluate evidence-based CPG interventions in LMICs. Established IS frameworks used in more than one study were the Exploration, Preparation, Implementation, and Sustainment (EPIS) framework (n = 2) and Consolidated Framework for Implementation Research (CFIR) (n = 2).

**Conclusions:**

There is limited research utilizing IS frameworks and strategies to promote CPG adoption and adherence in LMICs and substantial heterogeneity in reporting. Greater utilization of IS frameworks, strengthening implementation capacity and improving consistency in reporting, are needed to improve CPG uptake in LMICs.

**Systematic review registration:**

Open Science Framework (https://osf.io/nb75s).

## Background

Clinical practice guidelines (CPGs) can have a profound impact on cancer care. CPGs synthesize research, provide a standard of care, distill options when there is mixed evidence, promote shared decision-making, and help reduce practice variation between healthcare providers [1]. While resource-intensive to create, these systematically developed recommendations greatly assist and empower clinicians to provide evidenced-based care [2, 3].

Adoption of and adherence to cancer-specific CPGs, such as those published by the National Comprehensive Cancer Network (NCCN) and the American Society for Clinical Oncology (ASCO), have been linked to improved quality of care and increased survival [1–3]. Advancing evidence-based oncology care requires a multifaceted approach to move beyond creation of CPGs toward effective translation into routine clinical practice [4–6]. Even in high-income countries (HICs), the implementation of CPGs is not uniform. Multilevel barriers to guideline implementation include personal factors (e.g., lack of provider awareness), guideline-related factors (e.g., complexity), and health system factors (e.g., lack of resources) [2, 7].

As cancer incidence is projected to rise, with disproportionate disease burden in low- and middle-income countries (LMICs), optimizing treatment delivery is critical to improving survival disparities [8]. In LMICs, a lack of material resources and limited infrastructure are key barriers to optimizing cancer care delivery [9,10]. To mitigate these barriers, CPGs accounting for varying levels of resource availability have been created. This approach was pioneered by the Breast Health Global Initiative (BHGI) in the early 2000s and later adopted by the NCCN, ASCO, and other organizations to create resource-stratified, or harmonized, oncology CPGs [11–13].

Despite the existence of harmonized guidelines, transferring guidelines into clinical practice remains challenging [10, 12, 14, 15]. Implementation science (IS) research seeks to explore and address barriers to guideline uptake by utilizing structured implementation strategies [16]. Implementation *strategies* refer to *systematic processes* for integrating innovations and/or interventions into usual care [17]. In HICs, examples of effective strategies for guideline implementation include engaging providers through interactive education, clinical reminder systems, and periodic audit and feedback on guideline adherence [18, 19]. Strategies are often informed and guided by IS *frameworks*, which can be used to understand and explain the contextual factors that influence implementation outcomes and to assess the effectiveness of implementation efforts [20].

The use of IS frameworks and strategies to promote oncology CPG adoption and adherence in LMICs are limited. A 2016 scoping review of barriers to and strategies for guideline implementation included 69 articles, none of which represented research from an LMIC [7]. A subsequent 2023 systematic review of interventions to support healthcare providers’ compliance with guideline recommendations for breast cancer similarly did not include any publications from an LMIC [21]. Given the paucity of synthesized data on this topic, we initiated a scoping review to evaluate the state-of-the-literature regarding the application of IS frameworks and strategies to promote oncology CPG uptake in LMICs. Specifically, the aim of this scoping review is to identify use of IS frameworks and strategies to promote CPG adoption and adherence in LMICs. These aggregated results can be leveraged to improve CPG adoption and adherence in resource-limited settings globally.

## Methods

### Search strategy and eligibility criteria

This scoping review was performed following the Preferred Reporting Items for Systematic Reviews and Meta-Analyses extension for Scoping Reviews (PRISMA-ScR) guidelines [22]. The PRISMA-ScR checklist can be found in Supplementary material 1. A predefined protocol for this review was uploaded to Open Science Framework (OSF) on 03/18/2022 (https://osf.io/nb75s).

A medical librarian (LM) performed comprehensive searches to identify studies that describe, develop, reference, or utilize IS frameworks or strategies to deliver or evaluate cancer care in LMICs. Searches were conducted in the following databases on 03/21/2022: Medline (Ovid), Embase (Ovid), Cochrane Central Register of Controlled Trials (CENTRAL), African Journals Online (AJOL), Latin American and Caribbean Health Sciences Literature (LILACS), Scopus, Web of Science, and PsycINFO (Ovid). The search strategy included a wide array of terms for implementation, delivery, and evaluation of oncology guidelines in LMICs. The Cochrane Effective Practice and Organisation of Care Group’s LMIC Filter, based on the 2021 World Bank list of LMICs, was incorporated into the search to capture literature pertaining to implementation specifically in these countries [23]. The full search string can be found in Supplementary material 2. No date, language, or article type restrictions were included in the search strategy. Searches were re-run on 12/20/2022, 01/26/2024 and 10/03/2025 to account for any newly published studies missed in the initial search.

A grey literature search was also conducted across the following organizational websites to account for any literature not formally published in an academic journal (e.g. reports, government documents, policy papers): the World Health Organization (WHO), NCCN, BHGI, ASCO, the American Society of Breast Surgeons (ASBrS), the Society of Surgical Oncology (SSO), the European Society of Breast Cancer Specialists (EUSOMA), the African Organisation for Research and Training in Cancer (AORTIC), the Consortium of Universities for Global Health (CUGH), Academy Health, and the American Public Health Association (APHA). Grey literature search strategies can be found in Supplementary material 3.

Articles retrieved from search results were uploaded to Covidence systematic review software (Veritas Health Innovation, Melbourne, Australia), an internet-based systematic review management program, where collaborators reviewed abstracts and full texts during the study selection process. Each title and abstract were screened by two of seven independent reviewers (KL, OA, SM, JJ, BY, JO, CW) to minimize bias. Conflicts were resolved by a third reviewer (OO). Full-text screening was performed by two of six reviewers (AR, KL, WR, JO, CW, VM) who met to resolve conflicts.

Studies were included if they described, developed, referenced, or utilized an established or novel IS framework or strategy to evaluate or deliver cancer care in LMICs. Studies were excluded if they did not use (or clearly report using) an IS framework or strategy, or if the research was conducted in an HIC or did not focus on cancer care. Studies of all designs and publication types (e.g., randomized controlled trials, prospective studies, abstracts, meeting proceedings, letters to the editor, grey literature, etc.) were eligible for inclusion in this review. Duplicate study records (e.g., conference abstract and full-text manuscript of the same trial) were excluded.

### Data synthesis

Three reviewers (AR, KL, WR) independently performed data extraction in Covidence and met to resolve conflicts. Data extraction included: study title, first author, journal, year of publication, article type, CPG referenced, IS framework, implementation strategy, intervention target (i.e., patients, providers), implementation outcomes evaluated, method of evaluation, country where study took place, socioeconomic standing of country at time of study, total number of study participants, study aims, study design, cancer type, cancer stage (if applicable), clinical practice setting (i.e., primary, secondary, tertiary), and stage of cancer care continuum (i.e., prevention, early detection, diagnosis, treatment, palliative/supportive care, survivorship). Complete author lists were reviewed to determine whether any co-authors were based in the LMIC where the study took place. IS frameworks were further categorized into established or novel frameworks. Implementation strategies were initially mapped by one reviewer according to Proctor’s recommendations for specifying and reporting [24], and subsequently categorized according to Expert Recommendations for Implementing Change (ERIC) clusters [25]. Categorizations were reviewed by the study team, with discrepancies resolved by consensus. Implementation outcomes were reported as described by Proctor and colleagues [26]. Study characteristics were analyzed descriptively, and study results were summarized and categorized in data tables using a content analysis approach. In accordance with methodological guidance for scoping reviews, we did not conduct a quality assessment [22, 27].

## Results

### Article characteristics

**Fig. 1:**
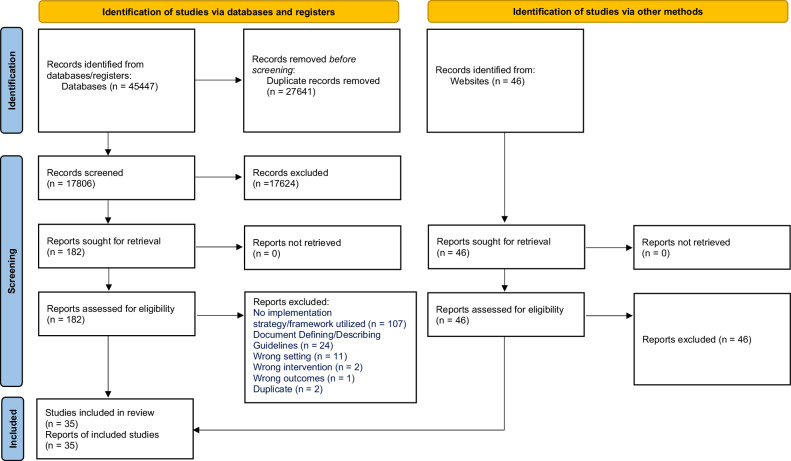
PRISMA diagram

Search strategies identified 17,806 unique publications for title/abstract screening (Fig. 1). Full-text review was performed on 182 studies, and 35 were included in the final data extraction (Table 1). Of the 147 excluded studies, 107 (73%) were excluded because they did not report using an IS framework or strategy.

**Table 1:** Characteristics of included studies (N = 35)

	N
**Geographic Scope**	
Single-country studies	24
Multi-country studies	11
Region (single-country studies only, n = 24)	
Sub-Saharan Africa (Ethiopia, Kenya, Malawi, Nigeria, Rwanda, Tanzania)	11
South Asia (Bangladesh, India)	6
Southeast Asia (Indonesia, Malaysia)	2
Latin America (Argentina, Peru)	3
East Asia (China)	1
Middle East (Iran)	1
**World Bank Lending Group**	
Low	3
Lower-middle	16
Upper-middle	5
**Publication year**	
2014-2018	4
2020-2025	31
**Cancer type**	
Cervical	14
Breast	8
Gynecologic	2
Pediatric- not specified	2
Bladder	1
Colon	1
Oral	1
All/not specified	8
**Article type**	
Original research manuscript	16
Review/commentary	8
Abstract/meeting proceeding	5
Protocol paper	4
Working group/consensus statement	2
**Study design**	
Review/commentary	8
Mixed methods	7
Observational	6
Quasi-experimental	5
Qualitative	3
Cluster randomized trial	2
Working group/consensus statement	2
Process evaluation	2

**Fig. 2:**
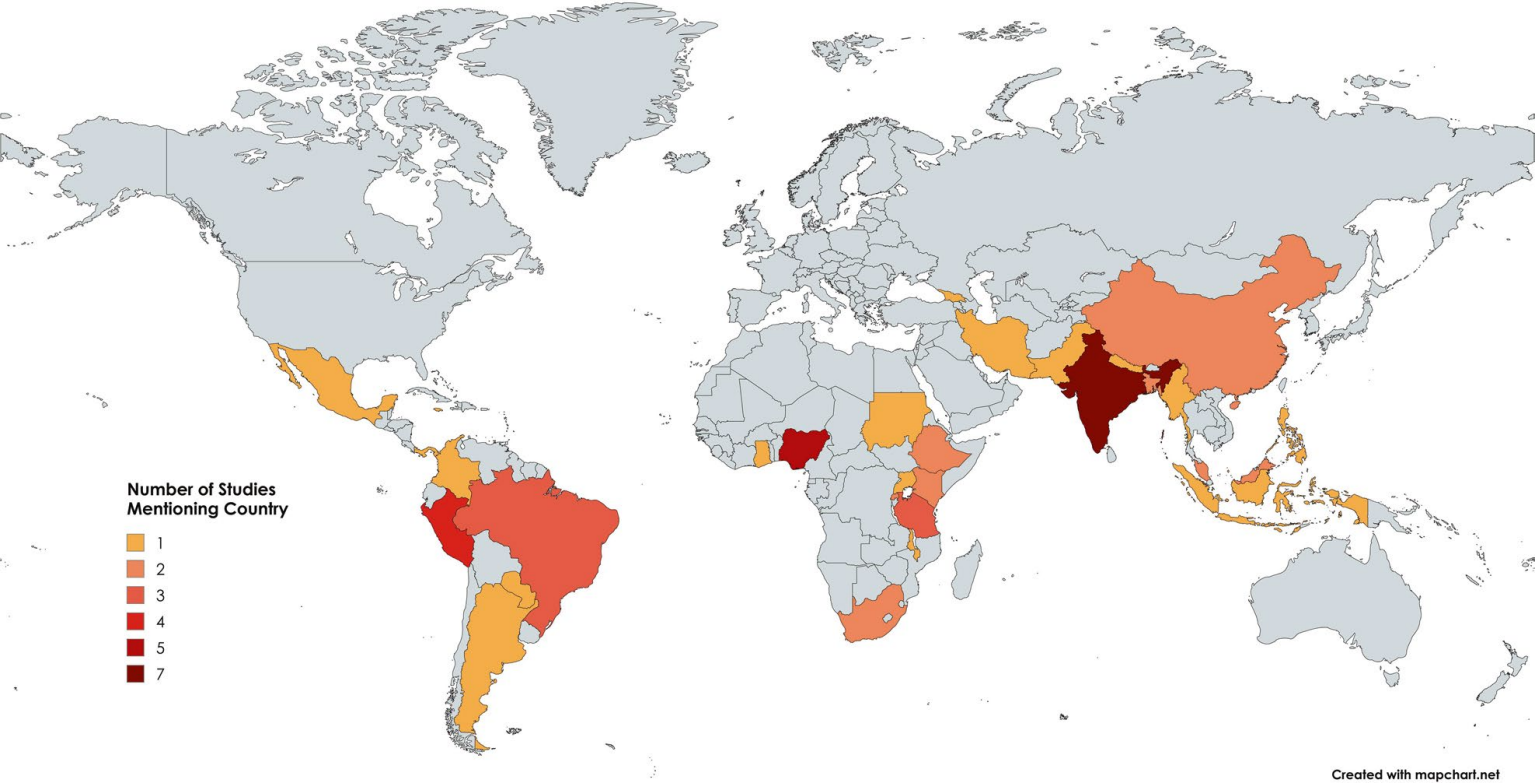
Countries represented

Most studies (n = 31/35, 89%) were published in 2020 or later. A map of countries represented is provided in Fig. 2. Twenty-four studies focused on a specific country, most commonly India (n = 5) and Nigeria (n = 5). While there were no language restrictions in the search, all publications had at least a translated abstract available for review and there was no literature in a language other than English that met inclusion criteria. Cervical (n = 14) and breast (n = 8) cancers were most frequently mentioned. The most frequent CPGs referenced were national/country-specific guidelines (n = 14) and BHGI guidelines (n = 10). Table 2 summarizes the CPGs referenced, IS frameworks, and outcomes of all 35 included studies.

**Table 2:** Clinical practice guidelines and implementation science frameworks, strategies, and outcomes of included studies (N = 35)

	N
Specific guidelines referenced*	
National/country-specific	14
Breast Health Global Initiative (BHGI)	10
World Health Organization (WHO) Guidelines for Cervical Cancer	5
National Comprehensive Cancer Network (NCCN)	4
American Society of Clinical Oncology (ASCO)	3
Oncology Nursing Society (ONS)	2
American Society of Clinical Pathology (ASCP)	1
American Society for Radiation Oncology (ASTRO)	1
Cancer Care Ontario	1
College of American Pathologists (CAP)	1
European Society of Medical Oncology	1
European Association of Urology Guidelines	1
Framework for Tobacco Control	1
International Atomic Energy Agency (IAEA)	1
International Society of Nurses in Cancer Care (ISNCC)	1
Saint Gallen International Consensus Conference	1
WHO Innovative Care for Chronic Conditions (ICCC) framework	1
WHO Clinical Nurse Educator Competencies	1
Developed a novel evidence-based Clinical Practice Guideline “Nursing Care of Cancer-Related Fatigue in Adults with Cancer”	1
None	11
**Intervention target** +	
Providers	23
Health system (representatives or policy)	12
Patients	4
**Implementation science frameworks**	
Existing frameworks	
Reach, Effectiveness, Adoption, Implementation, Maintenance (RE-AIM)	7
Consolidated Framework for Implementation Research (CFIR)	6
Exploration, Preparation, Implementation and Sustainment (EPIS)	6
Capability, Opportunity, Motivation and Behavior/Behavior Change Wheel (COM-B/BCW) Framework	2
Implementation Research Logic Model (IRLM)	2
Kirkpatrick Model	2
Assess-Couple-Test (ACT)	1
Capacity Mapping Framework	1
Conceptual Framework for Implementation Fidelity (CFIF)	1
Evidence Integration Triangle	1
Framework for Adaptations and Modifications-Expanded (FRAME)	1
Integrative Systems Praxis for Implementation Research (INSPIRE)	1
Interactive Systems Framework	1
Intervention Mapping Framework	1
Joanna Briggs Institute (JBI) Evidence Implementation Framework	1
JBI Getting Research into Practice (GRiP)	1
Mitchell and Chambers 2017 Framework	1
Model for Improvement	1
Problem Solving Cycle approach (PSC)	1
Promoting Action on Research Implementation in Health Services (PARIHS)	1
Quality Implementation Framework	1
Steps to Establish a National Education Program	1
Twinning model informed by the Partners in Health accompaniment approach	1
Villalobos Dintrans 2019 Framework	1
WHO Health Systems Core Building Blocks	1
Developed a novel framework	6
Novel frameworks developed	
Operational framework and resource-stratified care pathways for breast health services	2
National Cancer Control Plan (NCCP) Implementation Framework	1
Broad framework for assessing, planning and scaling up breast cancer early detection and treatment programs	1
City Engagement Process Framework (CEPF)	1
Framework for systematically evaluating cost and effectiveness metrics	1
**Implementation strategies (ERIC clusters** **)**	
Use evaluative and iterative strategies	31
Develop stakeholder interrelationships	24
Train and educate stakeholders	22
Adapt and tailor to context	22
Change infrastructure	12
Provide interactive assistance	12
Support clinicians	11
Engage consumers	11
Utilize financial strategies	8
**Implementation outcomes (Proctor** **)**	
Feasibility	20
Appropriateness	14
Acceptability	15
Adoption	17
Fidelity	14
Penetration	14
Implementation cost	11
Sustainability	8

*Some articles reference more than one guideline

+Some interventions have more than one target; 2 studies did not specify an intervention target

ERIC: Expert Recommendations for Implementing Change

Of the included 35 studies, only 16 were full-text, peer-reviewed, data-based manuscripts describing original research findings; the remaining studies were review/commentary (n = 8), abstracts/meeting proceedings (n = 5), protocols (n = 4), or working group/consensus statements (n = 2). Characteristics of the 16 full-text original research articles are detailed in Supplementary material 4 and summarized in the sections below. These 16 studies focused on cervical cancer screening/early detection (n = 7), breast healthcare services (n = 3, with two of the publications from the same line of research), improving the treatment of pediatric cancers (n = 2), post-discharge follow-up after bladder cancer surgery (n = 1), colorectal cancer screening (n = 1), cancer-related fatigue management (n = 1), and strengthening cancer care in cities (n = 1). At least one co-author on each of these 16 studies was based in an LMIC where the study was conducted.

### IS frameworks

Twelve of the 16 full-text original research articles described the use of an established IS framework. The Exploration, Preparation, Implementation, and Sustainment (EPIS) framework [28, 29] and the Consolidated Framework for Implementation Research (CFIR) were each used by 2 of the included studies [30–33]. Other frameworks included the Implementation Research Logic Model (IRLM) [34]; the Reach, Effectiveness, Adoption, Implementation, Maintenance (RE-AIM) framework [35–37]; the Promoting Action on Research Implementation in Health Services (PARIHS) framework [38–40]; the Capacity Mapping Framework [41, 42]; the Conceptual Framework for Implementation Fidelity (CFIF) [43, 44]; the Joanna Briggs Institute (JBI) Practical Application of Evidence System (PACES) framework; the JBI Getting Research into Practice (GRiP) [45–47]; the Kirkpatrick Model; the Model for Improvement; and the Problem Solving Cycle (PSC) [48–50].

Four studies developed a novel IS framework, including the City Engagement Process Framework [51], the National Cancer Control Plan (NCCP) Implementation Framework [52], and the operational framework and resource-stratified care pathways for breast health services [53, 54] (note: one study used established frameworks and developed a novel framework [51]).

### Implementation strategies

**Fig. 3:**
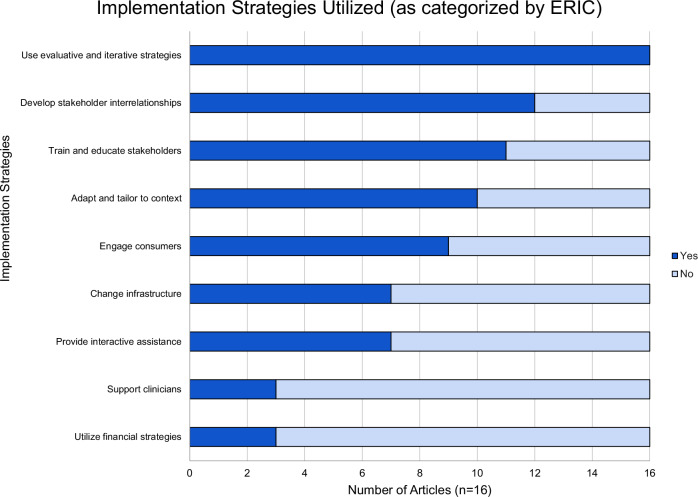
Implementation strategies summarized by Expert Recommendations for Implementing Change (ERIC) clusters

Because reporting of implementation strategies across studies had high variability [4], the implementation strategies used in the 16 full-text original research articles were categorized by ERIC clusters; these are detailed in Fig. 3 [17]. All 16 studies *used evaluative and iterative strategies* such as conducting local needs assessments, identifying barriers and facilitators, developing a formal implementation blueprint, developing tools for quality monitoring, performing audit and feedback, and purposely reexamining the implementation [30, 31, 37, 40, 42, 44, 47, 51, 54, 55]. Four studies used staged implementation scale up [31, 51, 53, 54]. One study used a formal pre-implementation application process including a thorough readiness assessment checklist [51].

Most studies (n = 11) *trained and educated stakeholders* often through development and distribution of educational materials and by conducting educational meetings [30, 31, 33, 37, 40, 44, 47, 49, 51, 53, 54]. Two studies used train-the-trainer strategies to implement cervical cancer screening [30, 44]. Most studies (n = 12) also made intentional efforts to *develop stakeholder interrelationships* to identify early adopters and potential implementation champions [30, 32, 33, 37, 40, 48–54]. The majority of studies (n = 10) *adapted and tailored* either the intervention itself or the implementation strategies to the local context [31, 32, 37, 40, 47–49, 51, 53, 54]. Almost half of the studies (n = 7) aimed to *provide interactive assistance* via facilitation [31, 37, 40, 48, 49, 53, 54] and to *change infrastructur*e [31, 44, 47, 50, 51, 53, 54] to promote implementation. Some of the studies (n = 9) further *engaged consumers* in the implementation process through patient or public health communication [30, 31, 37, 44, 49–51, 53, 54]. Fewer studies directly *supported clinicians* (n = 3) [31, 53, 54] or *utilized financial strategies* (n = 3) [31, 53, 54]. In addition to ERIC implementation strategies, one study developed 2 enhanced implementation strategies: 1) materials development and translation and 2) stakeholder/community engagement through Healthy Beginning Initiative (HBI) [30].

### Combining IS frameworks and implementation strategies

Several studies were especially rigorous in their application of established IS frameworks and strategies to enhance the design and/or execution of CPGs and other evidence-based intervention implementation. Itanyi and colleagues [30] employed a Rapid Implementation Mapping [56] process to identify implementation determinants, mechanisms, and strategies to implement evidence-based cervical cancer screening and treatment in existing human immunodeficiency virus (HIV) infrastructure in Nigeria. Engaging 11 key stakeholders, the investigators then used the EPIS framework to elicit discussion around determinants and strategies from the outer context (i.e., country and regions), inner organizational context of existing HIV infrastructure, and potential implementation strategies. Using this process, the team identified 18 determinants to successful integration of cervical cancer screening and treatment into existing comprehensive HIV programs, as well as 6 core and 2 enhanced implementation strategies [30]. Schliemann and colleagues [37] employed the IRLM to guide the design of a home-based colorectal cancer screening intervention in Malaysia. This intervention was then evaluated using the RE-AIM framework, via secondary data from annual health surveys, testing completion data, and qualitative interviews with a subset of participants (n = 48). From this evaluation, the team learned that only 22% of the target population (n = 311/1429) completed screening. However, by systematically using RE-AIM, the team was able to identify multiple barriers to reach, adoption, and acceptability, which will inform future work. Paolino and colleagues [44] evaluated fidelity to core components of the Self-collection Modality Trial (EMA, for its initials Spanish) human papillomavirus (HPV) self-collection strategy for cervical cancer screening in Argentina. Adherence to core components of this strategy, as proposed by the National Program on Cervical Cancer Prevention, were assessed using CFIF through a mixed-methods approach. The team identified core components with high fidelity and others that required local adaptation to maximize effectiveness [44]. Finally, Mallafre-Larrosa and colleagues [57] recently published a six-phase research protocol for the co-design, implementation and evaluation of a strategy to improve early cancer detection in rural India. During the pilot (phase 5) and scale-up (phase 6) stages, the authors aim to first apply the Practical, Robust Implementation and Sustainability Model (PRISM) framework to assess multilevel contextual factors that may impact implementation of the intervention, followed by the RE-AIM framework to evaluate intervention outcomes [57].

### Study design and outcomes measured

Guided by IS frameworks, the 16 full-text original research articles identified pre-implementation barriers and implementation process indicators (e.g., internal and external challenges to program implementation; readiness for implementation; stakeholder knowledge, attitudes, and behaviors; and situational analysis) and evaluated post-implementation outcomes (e.g., program adoption, fidelity to intervention content, intervention acceptability, and intervention feasibility) (Supplementary material 4). While most studies were observational, 3 studies used quasi-experimental study designs (e.g., pre- and post-test design). Implementation outcomes data were collected using qualitative (e.g., key informant interviews, focus group discussions), quantitative (e.g., surveys, chart reviews), and mixed-method (e.g., testing completion data and individual patient interviews) approaches. Four studies used a situational analysis approach, which involved interviews with key stakeholders and clinical audits [50, 51, 53, 54].

## Discussion

This scoping review sought to describe the state of the literature by examining the use of IS frameworks and strategies for promoting or evaluating adoption of oncology CPGs in LMICs. Overall, there is limited published research in this area. Of over 17,000 publications screened, only 16 full-text manuscripts of original research reported using an IS framework or strategy to inform the work (2 of which were from the same line of research). Twelve studies utilized established IS frameworks, whereas four utilized novel frameworks. All 16 full-text original research articles included a co-author based in the LMIC where the study took place, highlighting the importance of IS research led by investigators with contextual expertise.

These findings may reflect limited reporting of IS frameworks and strategies rather than definitive evidence that implementation activities are absent in LMICs, as such efforts may occur without being explicitly labeled or described using formal IS terminology. The absence of a reported IS framework or strategy was the predominant reason for article exclusion, accounting for over two-thirds (n = 107/147, 73%) of all excluded full-text articles. There is a growing, relevant IS literature illustrating the importance of using implementation strategies to understand and accelerate dissemination and implementation of CPGs [58, 59]. Implementation strategies guide the systematic integration of interventions into practice, while frameworks help to inform strategies and provide architecture for assessing contextual factors influencing outcomes of interest. IS frameworks offer the most potential benefit when used prior to and throughout the research [60]. This review highlights the limited integration and reporting of IS within oncology CPG implementation efforts in LMICs. However, the majority of included studies were published within the last 5 years, suggesting that increased attention is being paid to this important field in recent years. This review illustrates the need for further application of data-driven strategies to optimize the dissemination and implementation of CPGs in LMICs to promote intervention reach, fidelity, and sustainability.

There was heterogeneous reporting of implementation strategies across studies, which is seen as an obstacle for moving the field of adoption of CPGs in LMICs forward with greater rigor and reproducibility. When strategies were recategorized using ERIC clusters, we found that evaluative and iterative strategies were employed most commonly. Furthermore, deliberate efforts to develop stakeholder interrelationships and train/educate stakeholders were frequently used to promote sustainability of implementation efforts. Utilizing financial strategies was not often employed and highlights a gap to address with future interventions, particularly at a health policy level. Many studies identified were pre- or early implementation phase and focused on outcomes such as feasibility, appropriateness, acceptability, and adoption. Uniform reporting of IS frameworks, strategies, and outcomes in a way that is rigorous and reproducible could allow for better characterization of the IS literature and for easier comparison across studies and sites [24].

Of the 16 full-text original research articles, the only IS frameworks utilized in more than one study were the EPIS framework [28–31] and the Consolidated Framework for Implementation Research (CFIR) [32, 33,61, 62]. It is encouraging that stakeholders appreciated the value of considering EPIS stages of implementation. CFIR includes contextual domains that are particularly important in resource-limited settings (e.g., outer setting) and has been adapted for use in LMICs [63]. CFIR may be particularly well-suited for pre-implementation assessments and is recommended to be used to inform effective implementation strategies that are tailored to the LMIC context. IS frameworks that prioritize stakeholder engagement (e.g., implementation mapping framework) should be considered when adapting implementation strategies [56]. The importance of context-adapted methods in promoting utilization of CPGs in the LMIC setting cannot be overstated, as programs will likely need to adapt guidelines and implementation strategies to the local context while maintaining fidelity to the core elements [64].

The most frequently referenced oncology CPGs were tailored to local context: national/county-specific and resource-stratified (e.g., BHGI) guidelines. This suggests that most global oncology researchers understand that adaptation to the local context is critical for successful adoption and implementation of oncology CPGs. In contrast, studies from some LMIC settings suggest that while awareness and use of international oncology CPGs may be high, use of context-adapted or resource-stratified CPGs remains limited, highlighting the importance of continued research and specifically evaluation of tailored strategies to address this research-to-practice gap [65, 66].

Despite the limited number of empirical studies, there is some evidence that global oncology thought leaders have begun to consider dissemination and implementation of best practices. There were 11 review/commentary/consensus statement papers included in this review. Four of these were products of BHGI through their Global Summit on Improving Breast Healthcare through Resource-Stratified Phased Implementation in 2018 and were published in *Cancer* in 2020. In one of these publications, Rositch and colleagues detailed the role of IS in global breast cancer control programs [67]. Recommended implementation strategies included engaging multilevel stakeholders, collecting data from individuals up to systems level to inform policy and its dissemination/implementation, conducting formative research regarding the local context, utilizing implementation strategies to undertake action, and adapting an evidence-based intervention. The three other BHGI articles described phased implementation of breast healthcare, treatment, and early detection [5,68, 69]. Phased implementation was defined as “a process by which policymakers and implementers should first consider framing breast cancer control, through an initial situational analysis of the breast healthcare system, where breast cancer care is contextualized within the existing health system, followed by development of a sustainable strategy for strengthening breast healthcare systems via health system integration and financing schemes” [5]. Two additional commentary articles described the importance of provider education and training in oncology nurse educator mentorship and in palliative care [70, 71]. Cost/financial constraints must also be considered when implementing cancer control programs [72]. Other review articles focused on cancer prevention and control in LMICs, mobile health (mHealth) interventions to support cancer diagnosis in sub-Saharan Africa, and cancer treatment and palliative care strategies in LMICs [73–76].

One protocol paper identified in this review is especially rigorous in its study design for future research. DeBoer and colleagues used the Intervention Mapping framework to guide development of a theory-driven guideline implementation strategy at a single institution in Tanzania. Through a well-described process, this research integrated an established framework from its inception and engaged stakeholders to inform development, implementation, and evaluation of their strategy [16]. This application of IS is likely to accelerate translation of evidence into practice, promote uptake of CPGs in LMICs, and move the field of IS forward.

This scoping review has several strengths, including use of robust search strategies and query of multiple search engines under the guidance of a reference librarian. Publications in all languages and of all study types were eligible for inclusion, allowing for a broad and diverse group of included studies, which is particularly important in this emerging line of research. We also performed a grey literature review. Although none of the grey literature qualified for inclusion, this broadened our search, as we aimed to minimize the influence of bias in research being published from particular countries or regions. We were constrained by the limited reporting of IS frameworks and strategies in the studies evaluated. Studies were excluded if they did not report their methods in a way that was rigorous, reproducible, and consistent with IS literature. Our ability to categorize results and perform a quality appraisal of the literature was limited by the significant heterogeneity of the included studies.

## Conclusions

There is limited research utilizing IS frameworks and strategies to promote CPG adoption and adherence in LMICs and substantial heterogeneity in reporting. Strengthening implementation capacity and improving consistency in reporting are needed to accelerate and improve CPG uptake in LMICs. We make the following recommendations to strengthen adoption of oncology CPGs in LMICs:

1. Invest in IS training and collaborative opportunities for researchers based in LMICs.
2. Utilize IS frameworks throughout the implementation process; consider frameworks that prioritize context and stakeholder engagement.
3. Adapt implementation strategies to the local context while maintaining fidelity to the core elements.
4. Report IS frameworks and strategies in a way that is rigorous and reproducible.
5. Incentivize sustained adoption of oncology CPGs at the facility and health system levels, including through institutional performance metrics and organizational quality improvement initiatives.

Shortly after publication of the early resource-stratified oncology CPGs [11], an editorial pointed out that there was limited research on guideline implementation conducted in LMICs [77]. In the nearly two decades that followed, there has been greater attention drawn to this important topic, but progress remains slow. It is imperative to prioritize the application and reporting of IS frameworks and strategies to promote adoption of oncology CPGs in LMICs to standardize delivery of evidence-based care and improve patient outcomes.

## Supplementary information

Supplementary material 1: PRISMA-ScR checklist

Supplementary material 2: Search strategies

Supplementary material 3: Grey literature searches

Supplementary material 4: Full-text original research articles

## Data Availability

All data used in this study are included in this published article and its Supplementary materials.

## Abbreviations

AJOL: African Journals Online
AORTIC: African Organisation for Research and Training in Cancer
APHA: American Public Health Association
ASBrS: American Society of Breast Surgeons
ASCO: American Society for Clinical Oncology
BHGI: Breast Health Global Initiative
CENTRAL: Cochrane Central Register of Controlled Trials
CFIF: Conceptual Framework for Implementation Fidelity
CPG: clinical practice guideline
CUGH: Consortium of Universities for Global Health
EMA: Self-collection Modality Trial (for its initials EMA in Spanish)
EPIS: Exploration, Preparation, Implementation, and Sustainment
ERIC: Expert Recommendations for Implementing Change
EUSOMA: European Society of Breast Cancer Specialists
GRiP: Getting Research into Practice
HBI: Healthy Beginning Initiative
HIC: high-income country
HPV: human papillomavirus
IRLM: Implementation Research Logic Model
IS: implementation science
JBI: Joanna Briggs Institute
LILACS: Latin American and Caribbean Health Sciences Literature
LMIC: low- and middle-income country
NCCN: National Comprehensive Cancer Network
OSF: Open Science Framework
PACES: Practical Application of Evidence System
PARIHS: Promoting Action on Research Implementation in Health Services
PRISM: Practical, Robust Implementation and Sustainability Model
PRISMA-ScR: Preferred Reporting Items for Systematic Reviews and Meta-Analyses extension for Scoping Reviews
RE-AIM: Reach, Effectiveness, Adoption, Implementation, Maintenance
SSO: Society of Surgical Oncology
WHO: World Health Organization
