# Supplementary material for: Implementation science frameworks and strategies to promote adoption of and adherence to oncology clinical practice guidelines in low- and middle-income countries: a scoping review
Source: BMC Glob Public Health. 2026 Jul 16;4:67. doi: 10.1186/s44263-026-00297-4 (PMC13378326; doi:10.1186/s44263-026-00297-4)
Supplement: Supplementary file 2 — Supplementary material 2: Search strategies [file 44263_2026_297_MOESM2_ESM.docx]

**Supplementary material 2.** Search strategies.

# **Ovid MEDLINE(R) and Epub Ahead of Print, In-Process, In-Data-Review & Other Non-Indexed Citations, Daily and Versions 1946 to March 18, 2022**

| 1 | Health Plan Implementation/ | 6628 |
| --- | --- | --- |
| 2 | Program Evaluation/ | 66493 |
| 3 | Diffusion of Innovation/ | 18258 |
| 4 | exp guideline/ | 36870 |
| 5 | guideline adherence/ | 34651 |
| 6 | Quality Improvement/ | 30510 |
| 7 | ((Implement* or "Intervention mapping" or "Intervention design" or "Implementation mapping" or CFIR or "Consolidated framework for implementation research" or "Re-aim" or "Reach effectiveness adoption implementation maintenance" or "ERIC framework" or "Expert recommendations for implementing change" or TDF or "Theoretical domains framework" or "needs assessment" or "needs assessments" or Feasibility or "program evaluation" or "program sustainability" or dissemination or Guideline* or "Quality Improvement" or "evidence-based practice") adj5 (Cancer* or oncolog* or neoplasm* or tumor* or tumour* or malignan* or carcinoma*)).tw. | 35830 |
| 8 | or/1-7 | 218849 |
| 9 | exp Neoplasms/ | 3645801 |
| 10 | Oncology Service, Hospital/ | 1519 |
| 11 | 9 or 10 | 3646372 |
| 12 | (afghanistan or albania or algeria or american samoa or angola or "antigua and barbuda" or antigua or barbuda or argentina or armenia or armenian or aruba or azerbaijan or bahrain or bangladesh or barbados or republic of belarus or belarus or byelarus or belorussia or byelorussian or belize or british honduras or benin or dahomey or bhutan or bolivia or "bosnia and herzegovina" or bosnia or herzegovina or botswana or bechuanaland or brazil or brasil or bulgaria or burkina faso or burkina fasso or upper volta or burundi or urundi or cabo verde or cape verde or cambodia or kampuchea or khmer republic or cameroon or cameron or cameroun or central african republic or ubangi shari or chad or chile or china or colombia or comoros or comoro islands or iles comores or mayotte or democratic republic of the congo or democratic republic congo or congo or zaire or costa rica or "cote d’ivoire" or "cote d’ ivoire" or cote divoire or cote d ivoire or ivory coast or croatia or cuba or cyprus or czech republic or czechoslovakia or djibouti or french somaliland or dominica or dominican republic or ecuador or egypt or united arab republic or el salvador or equatorial guinea or spanish guinea or eritrea or estonia or eswatini or swaziland or ethiopia or fiji or gabon or gabonese republic or gambia or "georgia (republic)" or georgian or ghana or gold coast or gibraltar or greece or grenada or guam or guatemala or guinea or guinea bissau or guyana or british guiana or haiti or hispaniola or honduras or hungary or india or indonesia or timor or iran or iraq or isle of man or jamaica or jordan or kazakhstan or kazakh or kenya or "democratic people’s republic of korea" or republic of korea or north korea or south korea or korea or kosovo or kyrgyzstan or kirghizia or kirgizstan or kyrgyz republic or kirghiz or laos or lao pdr or "lao people's democratic republic" or latvia or lebanon or lebanese republic or lesotho or basutoland or liberia or libya or libyan arab jamahiriya or lithuania or macau or macao or republic of north macedonia or macedonia or madagascar or malagasy republic or malawi or nyasaland or malaysia or malay federation or malaya federation or maldives or indian ocean islands or indian ocean or mali or malta or micronesia or federated states of micronesia or kiribati or marshall islands or nauru or northern mariana islands or palau or tuvalu or mauritania or mauritius or mexico or moldova or moldovian or mongolia or montenegro or morocco or ifni or mozambique or portuguese east africa or myanmar or burma or namibia or nepal or netherlands antilles or nicaragua or niger or nigeria or oman or muscat or pakistan or panama or papua new guinea or new guinea or paraguay or peru or philippines or philipines or phillipines or phillippines or poland or "polish people's republic" or portugal or portuguese republic or puerto rico or romania or russia or russian federation or ussr or soviet union or union of soviet socialist republics or rwanda or ruanda or samoa or pacific islands or polynesia or samoan islands or navigator island or navigator islands or "sao tome and principe" or saudi arabia or senegal or serbia or seychelles or sierra leone or slovakia or slovak republic or slovenia or melanesia or solomon island or solomon islands or norfolk island or norfolk islands or somalia or south africa or south sudan or sri lanka or ceylon or "saint kitts and nevis" or "st. kitts and nevis" or saint lucia or "st. lucia" or "saint vincent and the grenadines" or saint vincent or "st. vincent" or grenadines or sudan or suriname or surinam or dutch guiana or netherlands guiana or syria or syrian arab republic or tajikistan or tadjikistan or tadzhikistan or tadzhik or tanzania or tanganyika or thailand or siam or timor leste or east timor or togo or togolese republic or tonga or "trinidad and tobago" or trinidad or tobago or tunisia or turkey or turkmenistan or turkmen or uganda or ukraine or uruguay or uzbekistan or uzbek or vanuatu or new hebrides or venezuela or vietnam or viet nam or middle east or west bank or gaza or palestine or yemen or yugoslavia or zambia or zimbabwe or northern rhodesia or global south or africa south of the sahara or sub-saharan africa or subsaharan africa or africa, central or central africa or africa, northern or north africa or northern africa or magreb or maghrib or sahara or africa, southern or southern africa or africa, eastern or east africa or eastern africa or africa, western or west africa or western africa or west indies or indian ocean islands or caribbean or central america or latin america or "south and central america" or south america or asia, central or central asia or asia, northern or north asia or northern asia or asia, southeastern or southeastern asia or south eastern asia or southeast asia or south east asia or asia, western or western asia or europe, eastern or east europe or eastern europe or developing country or developing countries or developing nation? or developing population? or developing world or less developed countr* or less developed nation? or less developed population? or less developed world or lesser developed countr* or lesser developed nation? or lesser developed population? or lesser developed world or under developed countr* or under developed nation? or under developed population? or under developed world or underdeveloped countr* or underdeveloped nation? or underdeveloped population? or underdeveloped world or middle income countr* or middle income nation? or middle income population? or low income countr* or low income nation? or low income population? or lower income countr* or lower income nation? or lower income population? or underserved countr* or underserved nation? or underserved population? or underserved world or under served countr* or under served nation? or under served population? or under served world or deprived countr* or deprived nation? or deprived population? or deprived world or poor countr* or poor nation? or poor population? or poor world or poorer countr* or poorer nation? or poorer population? or poorer world or developing econom* or less developed econom* or lesser developed econom* or under developed econom* or underdeveloped econom* or middle income econom* or low income econom* or lower income econom* or low gdp or low gnp or low gross domestic or low gross national or lower gdp or lower gnp or lower gross domestic or lower gross national or lmic or lmics or third world or lami countr* or transitional countr* or emerging economies or emerging nation?).ti,ab,sh,kf. | 2189572 |
| 13 | (afghan or afghans or afghani or albanian? algerian? or american samoan? or angolan? or antiguan? or barbudan? or argentine? or argentinian? or argentinean? or armenian? or aruban? or azerbaijani? or bahraini? or bangladeshi? or bangalees or bajan? or belarusian? or byelorussian? or belizean? or beninese? or bhutanese or bolivian? or bosnian? or botswana or batswana or brazilian? or brasilian? or bulgarian? or burkinabe or burkinese or burundian? or cape verdean? or cabo verdean? or cambodian? or khmer or cameroonian? or central african? or chadian? or chilean? or chinese or colombian? or comorian? or congolese or costa rican? or ivorian? or croatian? or cuban? or cypriot? or czech? or djiboutian? or dominican? or ecuadorian? or egyptian? or salvadoran? or equatorial guinean? or equatoguinean? or eritrean? or estonian? or swazi? or swati? or ethiopian? or fijian or gabonese or gabonaise or gambian? or georgian? or ghanaian? or gibraltarian? or greek? or grenadian? or guamanian? or guatemalan? or guinean? or bissau guinean? or guyanese or haitian? or honduran? or hungarian? or indian? or indonesian? or iranian? or iraqian? or iraqi? or manx or jamaican? or jordanian? or kazakhstani? or kenyan? or kirabati or kirabatian? or north korean? or korean? or kosovar? or kosovan? or kyrgyz* or lao or laotian? or latvian? or lebanese or lesothan? or lesothonian? or mosotho or basotho or liberian? or libyan? or lithuanian? or macanese or macedonian? or malagasy or madagascan? or malawian? or malaysian? or maldivian? or malian? or maltese or marshallese? or mauritanian? or mauritian? or mexican? or micronesian? or moldovan? or mongolian? or mongol or montenegrin? or moroccan? or mozambican? or burmese or myanma or namibian? or nauruan? or nepali or nepalese or netherlands antillean? or nicaraguan? or nigerien? or nigerian? or northern mariana islander? or mariana? or omani? or pakistani? or palauan? or panamanian? or papua new guinean? or paraguayan? or peruvian? or philippine? or philipine? or phillipine? or phillippine? or filipino? or filipina? or polish or pole or poles or portuguese or puerto rican? or romanian? or russian? or soviet people or soviet population or rwandan? or rwandese or ruandan? or ruandese or samoan? or sao tomean? or santomean? or saudi arabian? or saudi? or senegalese or serbian? or montenegrin? or seychellois or seychelloise? or sierra leonean? or slovak? or slovene? or solomon islander? or somali? or south african? or south sudanese or sri lankan? or ceylonese or kittitian? or nevisian? or saint lucian? or vincentian? or sudanese or surinamese? or syrian? or tajik? or tajikistani? or tanzanian? or tanganyikan? or thai or timorese? or togolese or tongan? or trinidadian? or tobagonian? or tunisian? or turk? or turkish or turkmen? or tuvaluan? or ugandan? or ukrainian? or uruguayan? or uzbek? or vanuatu* or venezuelan? or vietnamese or yemeni? or yemenite? or yemenese or yugoslav? or yugoslavian? or zambian? or zimbabwean?).ti,ab,sh,kf. | 965093 |
| 14 | 12 or 13 | 2596886 |
| 15 | 8 and 11 and 14 | 3045 |

# **Embase Classic+Embase 1947 to 2022 March 16**

| 1 | exp program evaluation/ | 30216 |
| --- | --- | --- |
| 2 | diffusion of innovation/ | 48 |
| 3 | practice guideline/ | 490603 |
| 4 | protocol compliance/ | 17692 |
| 5 | ((Implement* or "Intervention mapping" or "Intervention design" or "Implementation mapping" or CFIR or "Consolidated framework for implementation research" or "Re-aim" or "Reach effectiveness adoption implementation maintenance" or "ERIC framework" or "Expert recommendations for implementing change" or TDF or "Theoretical domains framework" or "needs assessment" or "needs assessments" or Feasibility or "program evaluation" or "program sustainability" or dissemination or Guideline* or "Quality Improvement" or "evidence-based practice") adj5 (Cancer* or oncolog* or neoplasm* or tumor* or tumour* or malignan* or carcinoma*)).tw. | 57695 |
| 6 | or/1-5 | 575705 |
| 7 | exp Neoplasm/ | 5400986 |
| 8 | cancer center/ | 44084 |
| 9 | 7 or 8 | 5410809 |
| 10 | (afghanistan or albania or algeria or american samoa or angola or "antigua and barbuda" or antigua or barbuda or argentina or armenia or armenian or aruba or azerbaijan or bahrain or bangladesh or barbados or republic of belarus or belarus or byelarus or belorussia or byelorussian or belize or british honduras or benin or dahomey or bhutan or bolivia or "bosnia and herzegovina" or bosnia or herzegovina or botswana or bechuanaland or brazil or brasil or bulgaria or burkina faso or burkina fasso or upper volta or burundi or urundi or cabo verde or cape verde or cambodia or kampuchea or khmer republic or cameroon or cameron or cameroun or central african republic or ubangi shari or chad or chile or china or colombia or comoros or comoro islands or iles comores or mayotte or democratic republic of the congo or democratic republic congo or congo or zaire or costa rica or "cote d’ivoire" or "cote d’ ivoire" or cote divoire or cote d ivoire or ivory coast or croatia or cuba or cyprus or czech republic or czechoslovakia or djibouti or french somaliland or dominica or dominican republic or ecuador or egypt or united arab republic or el salvador or equatorial guinea or spanish guinea or eritrea or estonia or eswatini or swaziland or ethiopia or fiji or gabon or gabonese republic or gambia or "georgia (republic)" or georgian or ghana or gold coast or gibraltar or greece or grenada or guam or guatemala or guinea or guinea bissau or guyana or british guiana or haiti or hispaniola or honduras or hungary or india or indonesia or timor or iran or iraq or isle of man or jamaica or jordan or kazakhstan or kazakh or kenya or "democratic people’s republic of korea" or republic of korea or north korea or south korea or korea or kosovo or kyrgyzstan or kirghizia or kirgizstan or kyrgyz republic or kirghiz or laos or lao pdr or "lao people's democratic republic" or latvia or lebanon or lebanese republic or lesotho or basutoland or liberia or libya or libyan arab jamahiriya or lithuania or macau or macao or republic of north macedonia or macedonia or madagascar or malagasy republic or malawi or nyasaland or malaysia or malay federation or malaya federation or maldives or indian ocean islands or indian ocean or mali or malta or micronesia or federated states of micronesia or kiribati or marshall islands or nauru or northern mariana islands or palau or tuvalu or mauritania or mauritius or mexico or moldova or moldovian or mongolia or montenegro or "montenegro (republic)" or morocco or ifni or mozambique or portuguese east africa or myanmar or burma or namibia or nepal or netherlands antilles or nicaragua or niger or nigeria or oman or muscat or pakistan or panama or papua new guinea or new guinea or paraguay or peru or philippines or philipines or phillipines or phillippines or poland or "polish people's republic" or portugal or portuguese republic or puerto rico or romania or russia or russian federation or ussr or soviet union or union of soviet socialist republics or rwanda or ruanda or samoa or pacific islands or polynesia or samoan islands or navigator island or navigator islands or "sao tome and principe" or saudi arabia or senegal or serbia or seychelles or sierra leone or slovakia or slovak republic or slovenia or melanesia or solomon island or solomon islands or norfolk island or norfolk islands or somalia or south africa or south sudan or sri lanka or ceylon or "saint kitts and nevis" or "st. kitts and nevis" or saint lucia or "st. lucia" or "saint vincent and the grenadines" or saint vincent or "st. vincent" or grenadines or sudan or suriname or surinam or dutch guiana or netherlands guiana or syria or syrian arab republic or tajikistan or tadjikistan or tadzhikistan or tadzhik or tanzania or tanganyika or thailand or siam or timor leste or east timor or togo or togolese republic or tonga or "trinidad and tobago" or trinidad or tobago or tunisia or "turkey (republic)" or turkey or turkmenistan or turkmen or uganda or ukraine or uruguay or uzbekistan or uzbek or vanuatu or new hebrides or venezuela or vietnam or viet nam or middle east or west bank or gaza or palestine or yemen or yugoslavia or zambia or zimbabwe or northern rhodesia or global south or africa south of the sahara or "sub saharan africa" or subsaharan africa or africa, central or central africa or africa, northern or north africa or northern africa or magreb or maghrib or sahara or africa, southern or southern africa or africa, eastern or east africa or eastern africa or africa, western or west africa or western africa or west indies or indian ocean islands or caribbean region or caribbean islands or caribbean or central america or latin america or "south and central america" or south america or asia, central or central asia or asia, northern or north asia or northern asia or asia, southeastern or southeastern asia or south eastern asia or southeast asia or south east asia or asia, western or western asia or europe, eastern or east europe or eastern europe or developing country or developing countries or developing nation? or developing population? or developing world or less developed countr* or less developed nation? or less developed population? or less developed world or lesser developed countr* or lesser developed nation? or lesser developed population? or lesser developed world or under developed countr* or under developed nation? or under developed population? or under developed world or underdeveloped countr* or underdeveloped nation? or underdeveloped population? or underdeveloped world or middle income countr* or middle income nation? or middle income population? or low income countr* or low income nation? or low income population? or lower income countr* or lower income nation? or lower income population? or underserved countr* or underserved nation? or underserved population? or underserved world or under served countr* or under served nation? or under served population? or under served world or deprived countr* or deprived nation? or deprived population? or deprived world or poor countr* or poor nation? or poor population? or poor world or poorer countr* or poorer nation? or poorer population? or poorer world or developing econom* or less developed econom* or lesser developed econom* or under developed econom* or underdeveloped econom* or middle income econom* or low income econom* or lower income econom* or low gdp or low gnp or low gross domestic or low gross national or lower gdp or lower gnp or lower gross domestic or lower gross national or lmic or lmics or third world or lami countr* or transitional countr* or emerging economies or emerging nation?).ti,ab,sh,kw. | 2642442 |
| 11 | (afghan or afghans or afghani or albanian? algerian? or american samoan? or angolan? or antiguan? or barbudan? or argentine? or argentinian? or argentinean? or armenian? or aruban? or azerbaijani? or bahraini? or bangladeshi? or bangalees or bajan? or belarusian? or byelorussian? or belizean? or beninese? or bhutanese or bolivian? or bosnian? or botswana or batswana or brazilian? or brasilian? or bulgarian? or burkinabe or burkinese or burundian? or cape verdean? or cabo verdean? or cambodian? or khmer or cameroonian? or central african? or chadian? or chilean? or chinese or colombian? or comorian? or congolese or costa rican? or ivorian? or croatian? or cuban? or cypriot? or czech? or djiboutian? or dominican? or ecuadorian? or egyptian? or salvadoran? or equatorial guinean? or equatoguinean? or eritrean? or estonian? or swazi? or swati? or ethiopian? or fijian or gabonese or gabonaise or gambian? or georgian? or ghanaian? or gibraltarian? or greek? or grenadian? or guamanian? or guatemalan? or guinean? or bissau guinean? or guyanese or haitian? or honduran? or hungarian? or indian? or indonesian? or iranian? or iraqian? or iraqi? or manx or jamaican? or jordanian? or kazakhstani? or kenyan? or kirabati or kirabatian? or north korean? or korean? or kosovar? or kosovan? or kyrgyz* or lao or laotian? or latvian? or lebanese or lesothan? or lesothonian? or mosotho or basotho or liberian? or libyan? or lithuanian? or macanese or macedonian? or malagasy or madagascan? or malawian? or malaysian? or maldivian? or malian? or maltese or marshallese? or mauritanian? or mauritian? or mexican? or micronesian? or moldovan? or mongolian? or mongol or montenegrin? or moroccan? or mozambican? or burmese or myanma or namibian? or nauruan? or nepali or nepalese or netherlands antillean? or nicaraguan? or nigerien? or nigerian? or northern mariana islander? or mariana? or omani? or pakistani? or palauan? or panamanian? or papua new guinean? or paraguayan? or peruvian? or philippine? or philipine? or phillipine? or phillippine? or filipino? or filipina? or polish or pole or poles or portuguese or puerto rican? or romanian? or russian? or soviet people or soviet population or rwandan? or rwandese or ruandan? or ruandese or samoan? or sao tomean? or santomean? or saudi arabian? or saudi? or senegalese or serbian? or montenegrin? or seychellois or seychelloise? or sierra leonean? or slovak? or slovene? or solomon islander? or somali? or south african? or south sudanese or sri lankan? or ceylonese or kittitian? or nevisian? or saint lucian? or vincentian? or sudanese or surinamese? or syrian? or tajik? or tajikistani? or tanzanian? or tanganyikan? or thai or timorese? or togolese or tongan? or trinidadian? or tobagonian? or tunisian? or turk? or turkish or turkmen? or tuvaluan? or ugandan? or ukrainian? or uruguayan? or uzbek? or vanuatu* or venezuelan? or vietnamese or yemeni? or yemenite? or yemenese or yugoslav? or yugoslavian? or zambian? or zimbabwean?).ti,ab,sh,kw. | 1350303 |
| 12 | 10 or 11 | 3353811 |
| 13 | 6 and 9 and 12 | 8531 |

# **APA PsycInfo 1806 to March Week 2 2022**

| 1 | Program Evaluation/ | 13309 |
| --- | --- | --- |
| 2 | Treatment Guidelines/ | 8247 |
| 3 | ((Implement* or "Intervention mapping" or "Intervention design" or "Implementation mapping" or CFIR or "Consolidated framework for implementation research" or "Re-aim" or "Reach effectiveness adoption implementation maintenance" or "ERIC framework" or "Expert recommendations for implementing change" or TDF or "Theoretical domains framework" or "needs assessment" or "needs assessments" or Feasibility or "program evaluation" or "program sustainability" or dissemination or Guideline* or "Quality Improvement" or "evidence-based practice") adj5 (Cancer* or oncolog* or neoplasm* or tumor* or tumour* or malignan* or carcinoma*)).tw. | 1755 |
| 4 | or/1-3 | 23120 |
| 5 | exp Neoplasms/ | 56728 |
| 6 | (afghanistan or albania or algeria or american samoa or angola or "antigua and barbuda" or antigua or barbuda or argentina or armenia or armenian or aruba or azerbaijan or bahrain or bangladesh or barbados or republic of belarus or belarus or byelarus or belorussia or byelorussian or belize or british honduras or benin or dahomey or bhutan or bolivia or "bosnia and herzegovina" or bosnia or herzegovina or botswana or bechuanaland or brazil or brasil or bulgaria or burkina faso or burkina fasso or upper volta or burundi or urundi or cabo verde or cape verde or cambodia or kampuchea or khmer republic or cameroon or cameron or cameroun or central african republic or ubangi shari or chad or chile or china or colombia or comoros or comoro islands or iles comores or mayotte or democratic republic of the congo or democratic republic congo or congo or zaire or costa rica or "cote d’ivoire" or "cote d’ ivoire" or cote divoire or cote d ivoire or ivory coast or croatia or cuba or cyprus or czech republic or czechoslovakia or djibouti or french somaliland or dominica or dominican republic or ecuador or egypt or united arab republic or el salvador or equatorial guinea or spanish guinea or eritrea or estonia or eswatini or swaziland or ethiopia or fiji or gabon or gabonese republic or gambia or "georgia (republic)" or georgian or ghana or gold coast or gibraltar or greece or grenada or guam or guatemala or guinea or guinea bissau or guyana or british guiana or haiti or hispaniola or honduras or hungary or india or indonesia or timor or iran or iraq or isle of man or jamaica or jordan or kazakhstan or kazakh or kenya or "democratic people’s republic of korea" or republic of korea or north korea or south korea or korea or kosovo or kyrgyzstan or kirghizia or kirgizstan or kyrgyz republic or kirghiz or laos or lao pdr or "lao people's democratic republic" or latvia or lebanon or lebanese republic or lesotho or basutoland or liberia or libya or libyan arab jamahiriya or lithuania or macau or macao or republic of north macedonia or macedonia or madagascar or malagasy republic or malawi or nyasaland or malaysia or malay federation or malaya federation or maldives or indian ocean islands or indian ocean or mali or malta or micronesia or federated states of micronesia or kiribati or marshall islands or nauru or northern mariana islands or palau or tuvalu or mauritania or mauritius or mexico or moldova or moldovian or mongolia or montenegro or morocco or ifni or mozambique or portuguese east africa or myanmar or burma or namibia or nepal or netherlands antilles or nicaragua or niger or nigeria or oman or muscat or pakistan or panama or papua new guinea or new guinea or paraguay or peru or philippines or philipines or phillipines or phillippines or poland or "polish people's republic" or portugal or portuguese republic or puerto rico or romania or russia or russian federation or ussr or soviet union or union of soviet socialist republics or rwanda or ruanda or samoa or pacific islands or polynesia or samoan islands or navigator island or navigator islands or "sao tome and principe" or saudi arabia or senegal or serbia or seychelles or sierra leone or slovakia or slovak republic or slovenia or melanesia or solomon island or solomon islands or norfolk island or norfolk islands or somalia or south africa or south sudan or sri lanka or ceylon or "saint kitts and nevis" or "st. kitts and nevis" or saint lucia or "st. lucia" or "saint vincent and the grenadines" or saint vincent or "st. vincent" or grenadines or sudan or suriname or surinam or dutch guiana or netherlands guiana or syria or syrian arab republic or tajikistan or tadjikistan or tadzhikistan or tadzhik or tanzania or tanganyika or thailand or siam or timor leste or east timor or togo or togolese republic or tonga or "trinidad and tobago" or trinidad or tobago or tunisia or turkey or turkmenistan or turkmen or uganda or ukraine or uruguay or uzbekistan or uzbek or vanuatu or new hebrides or venezuela or vietnam or viet nam or middle east or west bank or gaza or palestine or yemen or yugoslavia or zambia or zimbabwe or northern rhodesia or global south or africa south of the sahara or sub-saharan africa or subsaharan africa or africa, central or central africa or africa, northern or north africa or northern africa or magreb or maghrib or sahara or africa, southern or southern africa or africa, eastern or east africa or eastern africa or africa, western or west africa or western africa or west indies or indian ocean islands or caribbean or central america or latin america or "south and central america" or south america or asia, central or central asia or asia, northern or north asia or northern asia or asia, southeastern or southeastern asia or south eastern asia or southeast asia or south east asia or asia, western or western asia or europe, eastern or east europe or eastern europe or developing country or developing countries or developing nation? or developing population? or developing world or less developed countr* or less developed nation? or less developed population? or less developed world or lesser developed countr* or lesser developed nation? or lesser developed population? or lesser developed world or under developed countr* or under developed nation? or under developed population? or under developed world or underdeveloped countr* or underdeveloped nation? or underdeveloped population? or underdeveloped world or middle income countr* or middle income nation? or middle income population? or low income countr* or low income nation? or low income population? or lower income countr* or lower income nation? or lower income population? or underserved countr* or underserved nation? or underserved population? or underserved world or under served countr* or under served nation? or under served population? or under served world or deprived countr* or deprived nation? or deprived population? or deprived world or poor countr* or poor nation? or poor population? or poor world or poorer countr* or poorer nation? or poorer population? or poorer world or developing econom* or less developed econom* or lesser developed econom* or under developed econom* or underdeveloped econom* or middle income econom* or low income econom* or lower income econom* or low gdp or low gnp or low gross domestic or low gross national or lower gdp or lower gnp or lower gross domestic or lower gross national or lmic or lmics or third world or lami countr* or transitional countr* or emerging economies or emerging nation?).ti,ab,sh. | 249531 |
| 7 | (afghan or afghans or afghani or albanian? algerian? or american samoan? or angolan? or antiguan? or barbudan? or argentine? or argentinian? or argentinean? or armenian? or aruban? or azerbaijani? or bahraini? or bangladeshi? or bangalees or bajan? or belarusian? or byelorussian? or belizean? or beninese? or bhutanese or bolivian? or bosnian? or botswana or batswana or brazilian? or brasilian? or bulgarian? or burkinabe or burkinese or burundian? or cape verdean? or cabo verdean? or cambodian? or khmer or cameroonian? or central african? or chadian? or chilean? or chinese or colombian? or comorian? or congolese or costa rican? or ivorian? or croatian? or cuban? or cypriot? or czech? or djiboutian? or dominican? or ecuadorian? or egyptian? or salvadoran? or equatorial guinean? or equatoguinean? or eritrean? or estonian? or swazi? or swati? or ethiopian? or fijian or gabonese or gabonaise or gambian? or georgian? or ghanaian? or gibraltarian? or greek? or grenadian? or guamanian? or guatemalan? or guinean? or bissau guinean? or guyanese or haitian? or honduran? or hungarian? or indian? or indonesian? or iranian? or iraqian? or iraqi? or manx or jamaican? or jordanian? or kazakhstani? or kenyan? or kirabati or kirabatian? or north korean? or korean? or kosovar? or kosovan? or kyrgyz* or lao or laotian? or latvian? or lebanese or lesothan? or lesothonian? or mosotho or basotho or liberian? or libyan? or lithuanian? or macanese or macedonian? or malagasy or madagascan? or malawian? or malaysian? or maldivian? or malian? or maltese or marshallese? or mauritanian? or mauritian? or mexican? or micronesian? or moldovan? or mongolian? or mongol or montenegrin? or moroccan? or mozambican? or burmese or myanma or namibian? or nauruan? or nepali or nepalese or netherlands antillean? or nicaraguan? or nigerien? or nigerian? or northern mariana islander? or mariana? or omani? or pakistani? or palauan? or panamanian? or papua new guinean? or paraguayan? or peruvian? or philippine? or philipine? or phillipine? or phillippine? or filipino? or filipina? or polish or pole or poles or portuguese or puerto rican? or romanian? or russian? or soviet people or soviet population or rwandan? or rwandese or ruandan? or ruandese or samoan? or sao tomean? or santomean? or saudi arabian? or saudi? or senegalese or serbian? or montenegrin? or seychellois or seychelloise? or sierra leonean? or slovak? or slovene? or solomon islander? or somali? or south african? or south sudanese or sri lankan? or ceylonese or kittitian? or nevisian? or saint lucian? or vincentian? or sudanese or surinamese? or syrian? or tajik? or tajikistani? or tanzanian? or tanganyikan? or thai or timorese? or togolese or tongan? or trinidadian? or tobagonian? or tunisian? or turk? or turkish or turkmen? or tuvaluan? or ugandan? or ukrainian? or uruguayan? or uzbek? or vanuatu* or venezuelan? or vietnamese or yemeni? or yemenite? or yemenese or yugoslav? or yugoslavian? or zambian? or zimbabwean?).ti,ab,sh. | 243771 |
| 8 | 6 or 7 | 403000 |
| 9 | 4 and 5 and 8 | 151 |

# **Cochrane CENTRAL**

| ID | Search | Hits |
| --- | --- | --- |
| #1 | MeSH descriptor: [Health Plan Implementation] this term only | 190 |
| #2 | MeSH descriptor: [Program Evaluation] this term only | 6397 |
| #3 | MeSH descriptor: [Diffusion of Innovation] this term only | 136 |
| #4 | MeSH descriptor: [Guideline] explode all trees | 0 |
| #5 | MeSH descriptor: [Guideline Adherence] this term only | 1124 |
| #6 | MeSH descriptor: [Quality Improvement] this term only | 803 |
| #7 | ((Implement* or "Intervention mapping" or "Intervention design" or "Implementation mapping" or CFIR or "Consolidated framework for implementation research" or "Re-aim" or "Reach effectiveness adoption implementation maintenance" or "ERIC framework" or "Expert recommendations for implementing change" or TDF or "Theoretical domains framework" or "needs assessment" or "needs assessments" or Feasibility or "program evaluation" or "program sustainability" or dissemination or Guideline* or "Quality Improvement" or "evidence-based practice") NEAR/5 (Cancer* or oncolog* or neoplasm* or tumor* or tumour* or malignan* or carcinoma*)):ti,ab,kw | 3894 |
| #8 | {OR #1-#7} | 12141 |
| #9 | MeSH descriptor: [Neoplasms] explode all trees | 86823 |
| #10 | MeSH descriptor: [Oncology Service, Hospital] this term only | 24 |
| #11 | #9 OR #10 | 86828 |
| #12 | ((afghanistan OR albania OR algeria OR "american samoa" OR angola OR "antigua and barbuda" OR antigua OR barbuda OR argentina OR armenia OR armenian OR aruba OR azerbaijan OR bahrain OR bangladesh OR barbados OR "republic of belarus" OR belarus OR byelarus OR belorussia OR byelorussian OR belize OR "british honduras" OR benin OR dahomey OR bhutan OR bolivia OR "bosnia and herzegovina" OR bosnia OR herzegovina OR botswana OR bechuanaland OR brazil OR brasil OR bulgaria OR "burkina faso" OR "burkina fasso" OR "upper volta" OR burundi OR urundi OR "cabo verde" OR "cape verde" OR cambodia OR kampuchea OR "khmer republic" OR cameroon OR cameron OR cameroun OR "central african republic" OR "ubangi shari" OR chad OR chile OR china OR colombia OR comoros OR "comoro islands" OR "iles comores" OR mayotte OR "democratic republic of the congo" OR "democratic republic congo" OR congo OR zaire OR "costa rica" OR "cote d’ivoire" OR "cote d’ ivoire" OR "cote divoire" OR "cote d ivoire" OR "ivory coast" OR croatia OR cuba OR cyprus OR "czech republic" OR czechoslovakia OR djibouti OR "french somaliland" OR dominica OR "dominican republic" OR ecuador OR egypt OR "united arab republic" OR "el salvador" OR "equatorial guinea" OR "spanish guinea" OR eritrea OR estonia OR eswatini OR swaziland OR ethiopia OR fiji OR gabon OR "gabonese republic" OR gambia OR "georgia (republic)" OR georgia OR georgian OR ghana OR "gold coast" OR gibraltar OR greece OR grenada OR guam OR guatemala OR guinea OR "guinea bissau" OR guyana OR "british guiana" OR haiti OR hispaniola OR honduras OR hungary OR india OR indonesia OR timor OR iran OR iraq OR "isle of man" OR jamaica OR jordan OR kazakhstan OR kazakh OR kenya OR "democratic people’s republic of korea" OR "republic of korea" OR north korea OR south korea OR korea OR kosovo OR kyrgyzstan OR kirghizia OR kirgizstan OR "kyrgyz republic" OR kirghiz OR laos OR "lao pdr" OR "lao people's democratic republic" OR latvia OR lebanon OR "lebanese republic" OR lesotho OR basutoland OR liberia OR libya OR "libyan arab jamahiriya" OR lithuania OR macau OR macao OR "republic of north macedonia" OR macedonia OR madagascar OR "malagasy republic" OR malawi OR nyasaland OR malaysia OR "malay federation" OR "malaya federation" OR maldives OR "indian ocean islands" OR "indian ocean" OR mali OR malta OR micronesia OR "federated states of micronesia" OR kiribati OR "marshall islands" OR nauru OR "northern mariana islands" OR palau OR tuvalu OR mauritania OR mauritius OR mexico OR moldova OR moldovian OR mongolia OR montenegro OR morocco OR ifni OR mozambique OR "portuguese east africa" OR myanmar OR burma OR namibia OR nepal OR "netherlands antilles" OR nicaragua OR niger OR nigeria OR oman OR muscat OR pakistan OR panama OR "papua new guinea" OR paraguay OR peru OR philippines OR philipines OR phillipines OR phillippines OR poland OR "polish people's republic" OR portugal OR "portuguese republic" OR "puerto rico" OR romania OR russia OR "russian federation" OR ussr OR "soviet union" OR "union of soviet socialist republics" OR rwanda OR ruanda OR samoa OR "pacific islands" OR polynesia OR "samoan islands" OR "navigator island" OR "navigator islands" OR "sao tome and principe" OR "saudi arabia" OR senegal OR serbia OR seychelles OR "sierra leone" OR slovakia OR "slovak republic" OR slovenia OR melanesia OR "solomon island" OR "solomon islands" OR "norfolk island" OR "norfolk islands" OR somalia OR "south africa" OR "south sudan" OR "sri lanka" OR ceylon OR "saint kitts and nevis" OR "st. kitts and nevis" OR "saint lucia" OR "st. lucia" OR "saint vincent and the grenadines" OR "saint vincent" OR "st. vincent" OR grenadines OR sudan OR suriname OR surinam OR "dutch guiana" OR "netherlands guiana" OR syria OR "syrian arab republic" OR tajikistan OR tadjikistan OR tadzhikistan OR tadzhik OR tanzania OR tanganyika OR thailand OR siam OR "timor leste" OR "east timor" OR togo OR "togolese republic" OR tonga OR "trinidad and tobago" OR trinidad OR tobago OR tunisia OR turkey OR turkmenistan OR turkmen OR uganda OR ukraine OR uruguay OR uzbekistan OR uzbek OR vanuatu OR "new hebrides" OR venezuela OR vietnam OR "viet nam" OR "middle east" OR "west bank" OR gaza OR palestine OR yemen OR yugoslavia OR zambia OR zimbabwe OR "northern rhodesia" OR "global south" OR "africa south of the sahara" OR "sub saharan africa" OR "subsaharan africa" OR "africa, central" OR "central africa" OR "africa, northern" OR "north africa" OR "northern africa" OR magreb OR maghrib OR sahara OR "africa, southern" OR "southern africa" OR "africa, eastern" OR "east africa" OR "eastern africa" OR "africa, western" OR "west africa" OR "western africa" OR "west indies" OR "indian ocean islands" OR caribbean OR "central america" OR "latin america" OR "south and central america" OR "south america" OR "asia, central" OR "central asia" OR "asia, northern" OR "north asia" OR "northern asia" OR "asia, southeastern" OR "southeastern asia" OR "south eastern asia" OR "southeast asia" OR "south east asia" OR "asia, western" OR "western asia" OR "europe, eastern" OR "east europe" OR "eastern europe" OR "developing country" OR "developing countries" OR "developing nation" OR "developing nations" OR "developing population" OR "developing populations" OR "developing world" OR "less developed country" OR "less developed countries" OR "less developed nation" OR "less developed nations" OR "less developed population" OR "less developed populations" OR "less developed world" OR "lesser developed country" OR "lesser developed countries" OR "lesser developed nation" OR "lesser developed nations" OR "lesser developed population" OR "lesser developed populations" OR "lesser developed world" OR "under developed country" OR "under developed countries" OR "under developed nation" OR "under developed nations" OR "under developed population" OR "under developed populations" OR "under developed world" OR "underdeveloped country" OR "underdeveloped countries" OR "underdeveloped nation" OR "underdeveloped nations" OR "underdeveloped population" OR "underdeveloped populations" OR "underdeveloped world" OR "middle income country" OR "middle income countries" OR "middle income nation" OR "middle income nations" OR "middle income population" OR "middle income populations" OR "low income country" OR "low income countries" OR "low income nation" OR "low income nations" OR "low income population" OR "low income populations" OR "lower income country" OR "lower income countries" OR "lower income nation" OR "lower income nations" OR "lower income population" OR "lower income populations" OR "underserved country" OR "underserved countries" OR "underserved nation" OR "underserved nations" OR "underserved population" OR "underserved populations" OR "underserved world" OR "under served country" OR "under served countries" OR "under served nation" OR "under served nations" OR "under served population" OR "under served populations" OR "under served world" OR "deprived country" OR "deprived countries" OR "deprived nation" OR "deprived nations" OR "deprived population" OR "deprived populations" OR "deprived world" OR "poor country" OR "poor countries" OR "poor nation" OR "poor nations" OR "poor population" OR "poor populations" OR "poor world" OR "poorer country" OR "poorer countries" OR "poorer nation" OR "poorer nations" OR "poorer population" OR "poorer populations" OR "poorer world" OR "developing economy" OR "developing economies" OR "less developed economy" OR "less developed economies" OR "lesser developed economy" OR "lesser developed economies" OR "under developed economy" OR "under developed economies" OR "underdeveloped economy" OR "underdeveloped economies" OR "middle income economy" OR "middle income economies" OR "low income economy" OR "low income economies" OR "lower income economy" OR "lower income economies" OR "low gdp" OR "low gnp" OR "low gross domestic" OR "low gross national" OR "lower gdp" OR "lower gnp" OR "lower gross domestic" OR "lower gross national" OR lmic OR lmics OR "third world" OR "lami country" OR "lami countries" OR "transitional country" OR "transitional countries" OR "emerging economies" OR "emerging nation" OR "emerging nations")):ti,ab,kw | 107838 |
| #13 | ((afghan OR afghans OR afghani OR albanian OR albanians OR algerian OR algerians OR "american samoan" OR "american samoans" OR angolan OR angolans OR antiguan OR antiguans OR barbudan OR berbudans OR argentine OR argentines OR argentinian OR argentinians OR argentinean OR argentineans OR armenian OR armenians OR aruban OR arubans OR azerbaijani OR azerbaijanis OR bahraini OR bahrainis OR bangladeshi OR bangladeshis OR bangalees OR bajan OR bajans OR belarusian OR belarusians OR byelorussian OR byelorussians OR belizean OR belizeans OR beninese OR benineses OR bhutanese OR bolivian OR bolivians OR bosnian OR bosnians OR botswana OR batswana OR brazilian OR brazilians OR brasilian OR brasilians OR bulgarian OR bulgarians OR burkinabe OR burkinese OR burundian OR burundians OR "cape verdean" OR "cape verdeans" OR "cabo verdean" OR "cabo verdeans" OR cambodian OR cambodians OR khmer OR cameroonian OR cameroonians OR "central african" OR "central africans" OR chadian OR chadians OR chilean OR chileans OR chinese OR colombian OR colombians OR comorian OR comorians OR congolese OR "costa rican" OR "costa ricans" OR ivorian OR ivorians OR croatian OR croatians OR cuban OR cubans OR cypriot OR cypriots OR czech OR czechs OR djiboutian OR djiboutians OR dominican OR dominicans OR ecuadorian OR ecuadorians OR egyptian OR egyptians OR salvadoran OR salvadorans OR "equatorial guinean" OR "equatorial guineans" OR equatoguinean OR equatoguineans OR eritrean OR eritreans OR estonian OR estonians OR swazi OR swazis OR swati OR swatis OR ethiopian OR ethiopians OR fijian OR fijians OR gabonese OR gabonaise OR gambian OR gambians OR georgian OR georgians OR ghanaian OR ghanaians OR gibraltarian OR gibraltarians OR greek OR greeks OR grenadian OR grenadians OR guamanian OR guamanians OR guatemalan OR guatemalans OR guinean OR guineans OR "bissau guinean" OR "bissau guineans" OR guyanese OR haitian OR haitians OR honduran OR hondurans OR hungarian OR hungarians OR indian OR indians OR indonesian OR indonesians OR iranian OR iranians OR iraqian OR iraqians OR iraqi OR iraqis OR manx OR jamaican OR jamaicans OR jordanian OR jordanians OR kazakhstani OR kazakhstanis OR kenyan OR kenyans OR kirabati OR kirabatian OR kirabatians OR "north korean" OR "north koreans" OR korean OR koreans OR kosovar OR kosovars OR kosovan OR kosovans OR kyrgyzstani OR kyrgyzstanis OR kyrgyz OR lao OR laotian OR laotians OR latvian OR latvians OR lebanese OR lesothan OR lesothans OR lesothonian OR lesothonians OR mosotho OR basotho OR liberian OR liberians OR libyan OR libyans OR lithuanian OR lithuanians OR macanese OR macedonian OR macedonians OR malagasy OR madagascan OR madagascans OR malawian OR malawians OR malaysian OR malaysians OR maldivian OR maldivians OR malian OR malians OR maltese OR marshallese OR marshalleses OR mauritanian OR mauritanians OR  mauritian OR mauritians OR mexican OR mexicans OR micronesian OR micronesians OR moldovan OR moldovans OR mongolian OR mongolians OR mongol OR montenegrin OR montenegrins OR moroccan OR moroccans OR mozambican OR mozambicans OR burmese OR myanma OR namibian OR namibians OR nauruan OR nauruans OR nepali OR nepalese OR "netherlands antillean" OR "netherlands antilleans" OR nicaraguan OR nicaraguans OR nigerien OR  nigeriens OR nigerian OR nigerians OR "northern mariana islander" OR "northern mariana islanders" OR mariana OR marianas OR omani OR omanis OR pakistani OR pakistanis OR palauan OR palauans OR panamanian OR panamanians OR "papua new guinean" OR "papua new guineans" OR paraguayan OR paraguayans OR peruvian OR peruvians OR philippine OR philippines OR philipine OR philipines OR phillipine OR phillipines OR phillippine OR phillippines OR filipino OR filipinos OR filipina OR filipinas OR polish OR pole OR poles OR portuguese OR "puerto rican" OR "puerto ricans" OR romanian OR romanians OR russian OR russians OR "soviet people" OR "soviet population" OR rwandan OR rwandans OR rwandese OR ruandan OR ruandans OR ruandese OR samoan OR samoans OR "sao tomean" OR "sao tomeans" OR santomean OR santomeans OR "saudi arabian" OR "saudi arabians" OR saudi OR saudis OR senegalese OR serbian OR serbians OR montenegrin OR montenegrins OR seychellois OR seychelloise OR seychelloises OR "sierra leonean" OR "sierra leoneans" OR slovak OR slovaks OR slovene OR slovenes OR "solomon islander" OR "solomon islanders" OR somali OR somalis OR "south african" OR "south africans" OR "south sudanese" OR "sri lankan" OR "sri lankans" OR ceylonese OR kittitian OR kittitians OR nevisian OR nevisians OR "saint lucian" OR "saint lucians" OR vincentian OR vincentians OR sudanese OR surinamese OR surinameses OR syrian OR syrians OR tajik OR tajiks OR tajikistani OR tajikistanis OR tanzanian OR tanzanians OR tanganyikan OR tanganyikans OR thai OR timorese OR timoreses OR togolese OR tongan OR tongans OR trinidadian OR trinidadians OR tobagonian OR tobagonians OR tunisian OR tunisians OR turk OR turks OR turkish OR turkmen OR  turkmens OR tuvaluan OR tuvaluans OR ugandan OR ugandans OR ukrainian OR ukrainians uruguayan OR uruguayans OR uzbek OR uzbeks OR vanuatu OR vanuatuan OR vanuatuans OR venezuelan OR venezuelans OR vietnamese OR yemeni OR yemenis OR yemenite OR yemenites OR yemenese OR yugoslav OR yugoslavs OR yugoslavian OR yugoslavians OR zambian OR zambians OR zimbabwean OR zimbabweans)):ti,ab,kw | 69999 |
| #14 | #12 OR #13 | 149464 |
| #15 | #8 AND #11 AND #14 in Trials | 158 |

# **Scopus: 3337 results**

**1^st^ search: 190 results**

TITLE-ABS-KEY ( ( implement*  OR  "Intervention mapping"  OR  "Intervention design"  OR  "Implementation mapping"  OR  cfir  OR  "Consolidated framework for implementation research"  OR  "Re-aim"  OR  "Reach effectiveness adoption implementation maintenance"  OR  "ERIC framework"  OR  "Expert recommendations for implementing change"  OR  tdf  OR  "Theoretical domains framework"  OR  "needs assessment"  OR  "needs assessments"  OR  feasibility  OR  "program evaluation"  OR  "program sustainability"  OR  dissemination  OR  guideline*  OR  "Quality Improvement"  OR  "evidence-based practice" )  W/5  ( cancer*  OR  oncolog*  OR  neoplasm*  OR  tumor*  OR  tumour*  OR  malignan*  OR  carcinoma* ) )  AND  TITLE-ABS-KEY ( ( afghanistan  OR  albania  OR  algeria  OR  "american samoa"  OR  angola  OR  "antigua and barbuda"  OR  antigua  OR  barbuda  OR  argentina  OR  armenia  OR  armenian  OR  aruba  OR  azerbaijan  OR  bahrain  OR  bangladesh  OR  barbados  OR  "republic of belarus"  OR  belarus  OR  byelarus  OR  belorussia  OR  byelorussian  OR  belize  OR  "british honduras"  OR  benin  OR  dahomey  OR  bhutan  OR  bolivia  OR  "bosnia and herzegovina"  OR  bosnia  OR  herzegovina  OR  botswana  OR  bechuanaland  OR  brazil  OR  brasil  OR  bulgaria  OR  "burkina faso"  OR  "burkina fasso"  OR  "upper volta"  OR  burundi  OR  urundi  OR  "cabo verde"  OR  "cape verde"  OR  cambodia  OR  kampuchea  OR  "khmer republic"  OR  cameroon  OR  cameron  OR  cameroun  OR  "central african republic"  OR  "ubangi shari"  OR  chad  OR  chile  OR  china  OR  colombia  OR  comoros  OR  "comoro islands"  OR  "iles comores"  OR  mayotte  OR  "democratic republic of the congo"  OR  "democratic republic congo"  OR  congo  OR  zaire  OR  "costa rica"  OR  "cote d'ivoire"  OR  "cote d' ivoire"  OR  "cote divoire"  OR  "cote d ivoire"  OR  "ivory coast"  OR  croatia  OR  cuba  OR  cyprus  OR  "czech republic"  OR  czechoslovakia  OR  djibouti  OR  "french somaliland"  OR  dominica  OR  "dominican republic"  OR  ecuador  OR  egypt  OR  "united arab republic"  OR  "el salvador"  OR  "equatorial guinea"  OR  "spanish guinea"  OR  eritrea  OR  estonia  OR  eswatini  OR  swaziland  OR  ethiopia  OR  fiji  OR  gabon  OR  "gabonese republic"  OR  gambia  OR  "georgia (republic)"  OR  georgia  OR  georgian  OR  ghana  OR  "gold coast"  OR  gibraltar  OR  greece  OR  grenada  OR  guam  OR  guatemala  OR  guinea  OR  "guinea bissau"  OR  guyana  OR  "british guiana"  OR  haiti  OR  hispaniola  OR  honduras  OR  hungary  OR  india  OR  indonesia  OR  timor  OR  iran  OR  iraq  OR  "isle of man"  OR  jamaica  OR  jordan  OR  kazakhstan  OR  kazakh  OR  kenya  OR  "democratic people's republic of korea"  OR  "republic of korea"  OR  north  AND  korea  OR  south  AND  korea  OR  korea  OR  kosovo  OR  kyrgyzstan  OR  kirghizia  OR  kirgizstan  OR  "kyrgyz republic"  OR  kirghiz  OR  laos  OR  "lao pdr"  OR  "lao people's democratic republic"  OR  latvia  OR  lebanon  OR  "lebanese republic"  OR  lesotho  OR  basutoland  OR  liberia  OR  libya  OR  "libyan arab jamahiriya"  OR  lithuania  OR  macau  OR  macao  OR  "republic of north macedonia"  OR  macedonia  OR  madagascar  OR  "malagasy republic"  OR  malawi  OR  nyasaland  OR  malaysia ) )

**2^nd^ search: 1518 results**

TITLE-ABS-KEY ( ( implement*  OR  "Intervention mapping"  OR  "Intervention design"  OR  "Implementation mapping"  OR  cfir  OR  "Consolidated framework for implementation research"  OR  "Re-aim"  OR  "Reach effectiveness adoption implementation maintenance"  OR  "ERIC framework"  OR  "Expert recommendations for implementing change"  OR  tdf  OR  "Theoretical domains framework"  OR  "needs assessment"  OR  "needs assessments"  OR  feasibility  OR  "program evaluation"  OR  "program sustainability"  OR  dissemination  OR  guideline*  OR  "Quality Improvement"  OR  "evidence-based practice" )  W/5  ( cancer*  OR  oncolog*  OR  neoplasm*  OR  tumor*  OR  tumour*  OR  malignan*  OR  carcinoma* ) )

AND  TITLE-ABS-KEY ( ( "malay federation"  OR  "malaya federation"  OR  maldives  OR  "indian ocean islands"  OR  "indian ocean"  OR  mali  OR  malta  OR  micronesia  OR  "federated states of micronesia"  OR  kiribati  OR  "marshall islands"  OR  nauru  OR  "northern mariana islands"  OR  palau  OR  tuvalu  OR  mauritania  OR  mauritius  OR  mexico  OR  moldova  OR  moldovian  OR  mongolia  OR  montenegro  OR  morocco  OR  ifni  OR  mozambique  OR  "portuguese east africa"  OR  myanmar  OR  burma  OR  namibia  OR  nepal  OR  "netherlands antilles"  OR  nicaragua  OR  niger  OR  nigeria  OR  oman  OR  muscat  OR  pakistan  OR  panama  OR  "papua new guinea"  OR  paraguay  OR  peru  OR  philippines  OR  philipines  OR  phillipines  OR  phillippines  OR  poland  OR  "polish people's republic"  OR  portugal  OR  "portuguese republic"  OR  "puerto rico"  OR  romania  OR  russia  OR  "russian federation"  OR  ussr  OR  "soviet union"  OR  "union of soviet socialist republics"  OR  rwanda  OR  ruanda  OR  samoa  OR  "pacific islands"  OR  polynesia  OR  "samoan islands"  OR  "navigator island"  OR  "navigator islands"  OR  "sao tome and principe"  OR  "saudi arabia"  OR  senegal  OR  serbia  OR  seychelles  OR  "sierra leone"  OR  slovakia  OR  "slovak republic"  OR  slovenia  OR  melanesia  OR  "solomon island"  OR  "solomon islands"  OR  "norfolk island"  OR  "norfolk islands"  OR  somalia  OR  "south africa"  OR  "south sudan"  OR  "sri lanka"  OR  ceylon  OR  "saint kitts and nevis"  OR  "st. kitts and nevis"  OR  "saint lucia"  OR  "st. lucia"  OR  "saint vincent and the grenadines"  OR  "saint vincent"  OR  "st. vincent"  OR  grenadines  OR  sudan  OR  suriname  OR  surinam  OR  "dutch guiana"  OR  "netherlands guiana"  OR  syria  OR  "syrian arab republic"  OR  tajikistan  OR  tadjikistan  OR  tadzhikistan  OR  tadzhik  OR  tanzania  OR  tanganyika  OR  thailand  OR  siam  OR  "timor leste"  OR  "east timor"  OR  togo  OR  "togolese republic"  OR  tonga  OR  "trinidad and tobago"  OR  trinidad  OR  tobago  OR  tunisia  OR  turkey  OR  turkmenistan  OR  turkmen  OR  uganda  OR  ukraine  OR  uruguay  OR  uzbekistan  OR  uzbek  OR  vanuatu  OR  "new hebrides"  OR  venezuela  OR  vietnam  OR  "viet nam"  OR  "middle east"  OR  "west bank"  OR  gaza  OR  palestine  OR  yemen  OR  yugoslavia  OR  zambia  OR  zimbabwe  OR  "northern rhodesia"  OR  "global south"  OR  "africa south of the sahara"  OR  "sub saharan africa"  OR  "subsaharan africa"  OR  "africa, central"  OR  "central africa"  OR  "africa, northern"  OR  "north africa"  OR  "northern africa"  OR  magreb  OR  maghrib  OR  sahara  OR  "africa, southern"  OR  "southern africa"  OR  "africa, eastern"  OR  "east africa"  OR  "eastern africa"  OR  "africa, western"  OR  "west africa"  OR  "western africa"  OR  "west indies"  OR  "indian ocean islands"  OR  caribbean  OR  "central america"  OR  "latin america"  OR  "south and central america"  OR  "south america"  OR  "asia, central"  OR  "central asia"  OR  "asia, northern"  OR  "north asia"  OR  "northern asia"  OR  "asia, southeastern"  OR  "southeastern asia"  OR  "south eastern asia"  OR  "southeast asia"  OR  "south east asia"  OR  "asia, western"  OR  "western asia"  OR  "europe, eastern"  OR  "east europe"  OR  "eastern europe"  OR  "developing country"  OR  "developing countries"  OR  "developing nation"  OR  "developing nations"  OR  "developing population"  OR  "developing populations"  OR  "developing world"  OR  "less developed country"  OR  "less developed countries"  OR  "less developed nation"  OR  "less developed nations"  OR  "less developed population"  OR  "less developed populations"  OR  "less developed world"  OR  "lesser developed country"  OR  "lesser developed countries"  OR  "lesser developed nation"  OR  "lesser developed nations"  OR  "lesser developed population"  OR  "lesser developed populations"  OR  "lesser developed world"  OR  "under developed country"  OR  "under developed countries"  OR  "under developed nation"  OR  "under developed nations"  OR  "under developed population"  OR  "under developed populations"  OR  "under developed world"  OR  "underdeveloped country"  OR  "underdeveloped countries"  OR  "underdeveloped nation"  OR  "underdeveloped nations"  OR  "underdeveloped population"  OR  "underdeveloped populations"  OR  "underdeveloped world"  OR  "middle income country"  OR  "middle income countries"  OR  "middle income nation"  OR  "middle income nations"  OR  "middle income population" ) )

**3^rd^ search: 266**

TITLE-ABS-KEY ( ( implement*  OR  "Intervention mapping"  OR  "Intervention design"  OR  "Implementation mapping"  OR  cfir  OR  "Consolidated framework for implementation research"  OR  "Re-aim"  OR  "Reach effectiveness adoption implementation maintenance"  OR  "ERIC framework"  OR  "Expert recommendations for implementing change"  OR  tdf  OR  "Theoretical domains framework"  OR  "needs assessment"  OR  "needs assessments"  OR  feasibility  OR  "program evaluation"  OR  "program sustainability"  OR  dissemination  OR  guideline*  OR  "Quality Improvement"  OR  "evidence-based practice" )  W/5  ( cancer*  OR  oncolog*  OR  neoplasm*  OR  tumor*  OR  tumour*  OR  malignan*  OR  carcinoma* ) )

AND  TITLE-ABS-KEY ( ( "middle income populations"  OR  "low income country"  OR  "low income countries"  OR  "low income nation"  OR  "low income nations"  OR  "low income population"  OR  "low income populations"  OR  "lower income country"  OR  "lower income countries"  OR  "lower income nation"  OR  "lower income nations"  OR  "lower income population"  OR  "lower income populations"  OR  "underserved country"  OR  "underserved countries"  OR  "underserved nation"  OR  "underserved nations"  OR  "underserved population"  OR  "underserved populations"  OR  "underserved world"  OR  "under served country"  OR  "under served countries"  OR  "under served nation"  OR  "under served nations"  OR  "under served population"  OR  "under served populations"  OR  "under served world"  OR  "deprived country"  OR  "deprived countries"  OR  "deprived nation"  OR  "deprived nations"  OR  "deprived population"  OR  "deprived populations"  OR  "deprived world"  OR  "poor country"  OR  "poor countries"  OR  "poor nation"  OR  "poor nations"  OR  "poor population"  OR  "poor populations"  OR  "poor world"  OR  "poorer country"  OR  "poorer countries"  OR  "poorer nation"  OR  "poorer nations"  OR  "poorer population"  OR  "poorer populations"  OR  "poorer world"  OR  "developing economy"  OR  "developing economies"  OR  "less developed economy"  OR  "less developed economies"  OR  "lesser developed economy"  OR  "lesser developed economies"  OR  "under developed economy"  OR  "under developed economies"  OR  "underdeveloped economy"  OR  "underdeveloped economies"  OR  "middle income economy"  OR  "middle income economies"  OR  "low income economy"  OR  "low income economies"  OR  "lower income economy"  OR  "lower income economies"  OR  "low gdp"  OR  "low gnp"  OR  "low gross domestic"  OR  "low gross national"  OR  "lower gdp"  OR  "lower gnp"  OR  "lower gross domestic"  OR  "lower gross national"  OR  lmic  OR  lmics  OR  "third world"  OR  "lami country"  OR  "lami countries"  OR  "transitional country"  OR  "transitional countries"  OR  "emerging economies"  OR  "emerging nation"  OR  "emerging nations" ) )

**4^th^ search: 1359**

TITLE-ABS-KEY ( ( implement*  OR  "Intervention mapping"  OR  "Intervention design"  OR  "Implementation mapping"  OR  cfir  OR  "Consolidated framework for implementation research"  OR  "Re-aim"  OR  "Reach effectiveness adoption implementation maintenance"  OR  "ERIC framework"  OR  "Expert recommendations for implementing change"  OR  tdf  OR  "Theoretical domains framework"  OR  "needs assessment"  OR  "needs assessments"  OR  feasibility  OR  "program evaluation"  OR  "program sustainability"  OR  dissemination  OR  guideline*  OR  "Quality Improvement"  OR  "evidence-based practice" )  W/5  ( cancer*  OR  oncolog*  OR  neoplasm*  OR  tumor*  OR  tumour*  OR  malignan*  OR  carcinoma* ) )

AND

TITLE-ABS-KEY ( ( afghan  OR  afghans  OR  afghani  OR  albanian  OR  albanians  OR  algerian  OR  algerians  OR  "american samoan"  OR  "american samoans"  OR  angolan  OR  angolans  OR  antiguan  OR  antiguans  OR  barbudan  OR  berbudans  OR  argentine  OR  argentines  OR  argentinian  OR  argentinians  OR  argentinean  OR  argentineans  OR  armenian  OR  armenians  OR  aruban  OR  arubans  OR  azerbaijani  OR  azerbaijanis  OR  bahraini  OR  bahrainis  OR  bangladeshi  OR  bangladeshis  OR  bangalees  OR  bajan  OR  bajans  OR  belarusian  OR  belarusians  OR  byelorussian  OR  byelorussians  OR  belizean  OR  belizeans  OR  beninese  OR  benineses  OR  bhutanese  OR  bolivian  OR  bolivians  OR  bosnian  OR  bosnians  OR  botswana  OR  batswana  OR  brazilian  OR  brazilians  OR  brasilian  OR  brasilians  OR  bulgarian  OR  bulgarians  OR  burkinabe  OR  burkinese  OR  burundian  OR  burundians  OR  "cape verdean"  OR  "cape verdeans"  OR  "cabo verdean"  OR  "cabo verdeans"  OR  cambodian  OR  cambodians  OR  khmer  OR  cameroonian  OR  cameroonians  OR  "central african"  OR  "central africans"  OR  chadian  OR  chadians  OR  chilean  OR  chileans  OR  chinese  OR  colombian  OR  colombians  OR  comorian  OR  comorians  OR  congolese  OR  "costa rican"  OR  "costa ricans"  OR  ivorian  OR  ivorians  OR  croatian  OR  croatians  OR  cuban  OR  cubans  OR  cypriot  OR  cypriots  OR  czech  OR  czechs  OR  djiboutian  OR  djiboutians  OR  dominican  OR  dominicans  OR  ecuadorian  OR  ecuadorians  OR  egyptian  OR  egyptians  OR  salvadoran  OR  salvadorans  OR  "equatorial guinean"  OR  "equatorial guineans"  OR  equatoguinean  OR  equatoguineans  OR  eritrean  OR  eritreans  OR  estonian  OR  estonians  OR  swazi  OR  swazis  OR  swati  OR  swatis  OR  ethiopian  OR  ethiopians  OR  fijian  OR  fijians  OR  gabonese  OR  gabonaise  OR  gambian  OR  gambians  OR  georgian  OR  georgians  OR  ghanaian  OR  ghanaians  OR  gibraltarian  OR  gibraltarians  OR  greek  OR  greeks  OR  grenadian  OR  grenadians  OR  guamanian  OR  guamanians  OR  guatemalan  OR  guatemalans  OR  guinean  OR  guineans  OR  "bissau guinean"  OR  "bissau guineans"  OR  guyanese  OR  haitian  OR  haitians  OR  honduran  OR  hondurans  OR  hungarian  OR  hungarians  OR  indian  OR  indians  OR  indonesian  OR  indonesians  OR  iranian  OR  iranians  OR  iraqian  OR  iraqians  OR  iraqi  OR  iraqis  OR  manx  OR  jamaican  OR  jamaicans  OR  jordanian  OR  jordanians  OR  kazakhstani  OR  kazakhstanis  OR  kenyan  OR  kenyans  OR  kirabati  OR  kirabatian  OR  kirabatians  OR  "north korean"  OR  "north koreans"  OR  korean  OR  koreans  OR  kosovar  OR  kosovars  OR  kosovan  OR  kosovans  OR  kyrgyzstani  OR  kyrgyzstanis  OR  kyrgyz  OR  lao  OR  laotian  OR  laotians  OR  latvian  OR  latvians  OR  lebanese  OR  lesothan  OR  lesothans  OR  lesothonian  OR  lesothonians  OR  mosotho  OR  basotho  OR  liberian  OR  liberians  OR  libyan  OR  libyans ) )

**5^th^ search: 4**

TITLE-ABS-KEY ( ( implement*  OR  "Intervention mapping"  OR  "Intervention design"  OR  "Implementation mapping"  OR  cfir  OR  "Consolidated framework for implementation research"  OR  "Re-aim"  OR  "Reach effectiveness adoption implementation maintenance"  OR  "ERIC framework"  OR  "Expert recommendations for implementing change"  OR  tdf  OR  "Theoretical domains framework"  OR  "needs assessment"  OR  "needs assessments"  OR  feasibility  OR  "program evaluation"  OR  "program sustainability"  OR  dissemination  OR  guideline*  OR  "Quality Improvement"  OR  "evidence-based practice" )  W/5  ( cancer*  OR  oncolog*  OR  neoplasm*  OR  tumor*  OR  tumour*  OR  malignan*  OR  carcinoma* ) )

AND

TITLE-ABS-KEY ( ( lithuanian  OR  lithuanians  OR  macanese  OR  macedonian  OR  macedonians  OR  malagasy  OR  madagascan  OR  madagascans  OR  malawian  OR  malawians  OR  malaysian  OR  malaysians  OR  maldivian  OR  maldivians  OR  malian  OR  malians  OR  maltese  OR  marshallese  OR  marshalleses  OR  mauritanian  OR  mauritanians  OR  mauritian  OR  mauritians  OR  mexican  OR  mexicans  OR  micronesian  OR  micronesians  OR  moldovan  OR  moldovans  OR  mongolian  OR  mongolians  OR  mongol  OR  montenegrin  OR  montenegrins  OR  moroccan  OR  moroccans  OR  mozambican  OR  mozambicans  OR  burmese  OR  myanma  OR  namibian  OR  namibians  OR  nauruan  OR  nauruans  OR  nepali  OR  nepalese  OR  "netherlands antillean"  OR  "netherlands antilleans"  OR  nicaraguan  OR  nicaraguans  OR  nigerien  OR  nigeriens  OR  nigerian  OR  nigerians  OR  "northern mariana islander"  OR  "northern mariana islanders"  OR  mariana  OR  marianas  OR  omani  OR  omanis  OR  pakistani  OR  pakistanis  OR  palauan  OR  palauans  OR  panamanian  OR  panamanians  OR  "papua new guinean"  OR  "papua new guineans"  OR  paraguayan  OR  paraguayans  OR  peruvian  OR  peruvians  OR  philippine  OR  philippines  OR  philipine  OR  philipines  OR  phillipine  OR  phillipines  OR  phillippine  OR  phillippines  OR  filipino  OR  filipinos  OR  filipina  OR  filipinas  OR  polish  OR  pole  OR  poles  OR  portuguese  OR  "puerto rican"  OR  "puerto ricans"  OR  romanian  OR  romanians  OR  russian  OR  russians  OR  "soviet people"  OR  "soviet population"  OR  rwandan  OR  rwandans  OR  rwandese  OR  ruandan  OR  ruandans  OR  ruandese  OR  samoan  OR  samoans  OR  "sao tomean"  OR  "sao tomeans"  OR  santomean  OR  santomeans  OR  "saudi arabian"  OR  "saudi arabians"  OR  saudi  OR  saudis  OR  senegalese  OR  serbian  OR  serbians  OR  montenegrin  OR  montenegrins  OR  seychellois  OR  seychelloise  OR  seychelloises  OR  "sierra leonean"  OR  "sierra leoneans"  OR  slovak  OR  slovaks  OR  slovene  OR  slovenes  OR  "solomon islander"  OR  "solomon islanders"  OR  somali  OR  somalis  OR  "south african"  OR  "south africans"  OR  "south sudanese"  OR  "sri lankan"  OR  "sri lankans"  OR  ceylonese  OR  kittitian  OR  kittitians  OR  nevisian  OR  nevisians  OR  "saint lucian"  OR  "saint lucians"  OR  vincentian  OR  vincentians  OR  sudanese  OR  surinamese  OR  surinameses  OR  syrian  OR  syrians  OR  tajik  OR  tajiks  OR  tajikistani  OR  tajikistanis  OR  tanzanian  OR  tanzanians  OR  tanganyikan  OR  tanganyikans  OR  thai  OR  timorese  OR  timoreses  OR  togolese  OR  tongan  OR  tongans  OR  trinidadian  OR  trinidadians  OR  tobagonian  OR  tobagonians  OR  tunisian  OR  tunisians  OR  turk  OR  turks  OR  turkish  OR  turkmen  OR  turkmens  OR  tuvaluan  OR  tuvaluans  OR  ugandan  OR  ugandans  OR  ukrainian  OR  ukrainians  AND uruguayan  OR  uruguayans  OR  uzbek  OR  uzbeks  OR  vanuatu  OR  vanuatuan  OR  vanuatuans  OR  venezuelan  OR  venezuelans  OR  vietnamese  OR  yemeni  OR  yemenis  OR  yemenite  OR  yemenites  OR  yemenese  OR  yugoslav  OR  yugoslavs  OR  yugoslavian  OR  yugoslavians  OR  zambian  OR  zambians  OR  zimbabwean  OR  zimbabweans ) )

# **Web of Science: 2489 results**

TS=((implement*  OR  "Intervention mapping"  OR  "Intervention design"  OR  "Implementation mapping"  OR  cfir  OR  "Consolidated framework for implementation research"  OR  "Re-aim"  OR  "Reach effectiveness adoption implementation maintenance"  OR  "ERIC framework"  OR  "Expert recommendations for implementing change"  OR  tdf  OR  "Theoretical domains framework"  OR  "needs assessment"  OR  "needs assessments"  OR  feasibility  OR  "program evaluation"  OR  "program sustainability"  OR  dissemination  OR  guideline*  OR  "Quality Improvement"  OR  "evidence-based practice" ) NEAR/5 (cancer* or neoplasm* or tumor* or tumour* or carcinoma*)) AND

TS=(afghanistan  OR albania  OR algeria  OR "american samoa"  OR angola  OR "antigua and barbuda"  OR antigua  OR barbuda  OR argentina  OR armenia  OR armenian  OR aruba  OR azerbaijan  OR bahrain  OR bangladesh  OR barbados  OR "republic of belarus"  OR belarus  OR byelarus  OR belorussia  OR byelorussian  OR belize  OR "british honduras"  OR benin  OR dahomey  OR bhutan  OR bolivia  OR "bosnia and herzegovina"  OR bosnia  OR herzegovina  OR botswana  OR bechuanaland  OR brazil  OR brasil  OR bulgaria  OR "burkina faso"  OR "burkina fasso"  OR "upper volta"  OR burundi  OR urundi  OR "cabo verde"  OR "cape verde"  OR cambodia  OR kampuchea  OR "khmer republic"  OR cameroon  OR cameron  OR cameroun  OR "central african republic"  OR "ubangi shari"  OR chad  OR chile  OR china  OR colombia  OR comoros  OR "comoro islands"  OR "iles comores"  OR mayotte  OR "democratic republic of the congo"  OR "democratic republic congo"  OR congo  OR zaire  OR "costa rica"  OR "cote divoire"  OR "cote d ivoire"  OR "cote divoire"  OR "cote d ivoire"  OR "ivory coast"  OR croatia  OR cuba  OR cyprus  OR "czech republic"  OR czechoslovakia  OR djibouti  OR "french somaliland"  OR dominica  OR "dominican republic"  OR ecuador  OR egypt  OR "united arab republic"  OR "el salvador"  OR "equatorial guinea"  OR "spanish guinea"  OR eritrea  OR estonia  OR eswatini  OR swaziland  OR ethiopia  OR fiji  OR gabon  OR "gabonese republic"  OR gambia  OR "georgia (republic)"  OR georgia  OR georgian  OR ghana  OR "gold coast"  OR gibraltar  OR greece  OR grenada  OR guam  OR guatemala  OR guinea  OR "guinea bissau"  OR guyana  OR "british guiana"  OR haiti  OR hispaniola  OR honduras  OR hungary  OR india  OR indonesia  OR timor  OR iran  OR iraq  OR "isle of man"  OR jamaica  OR jordan  OR kazakhstan  OR kazakh  OR kenya  OR "democratic peoples republic of korea"  OR "republic of korea"  OR north korea  OR south korea  OR korea  OR kosovo  OR kyrgyzstan  OR kirghizia  OR kirgizstan  OR "kyrgyz republic"  OR kirghiz  OR laos  OR "lao pdr"  OR "lao people's democratic republic"  OR latvia  OR lebanon  OR "lebanese republic"  OR lesotho  OR basutoland  OR liberia  OR libya  OR "libyan arab jamahiriya"  OR lithuania  OR macau  OR macao  OR "republic of north macedonia"  OR macedonia  OR madagascar  OR "malagasy republic"  OR malawi  OR nyasaland  OR malaysia  OR "malay federation"  OR "malaya federation"  OR maldives  OR "indian ocean islands"  OR "indian ocean"  OR mali  OR malta  OR micronesia  OR "federated states of micronesia"  OR kiribati  OR "marshall islands"  OR nauru  OR "northern mariana islands"  OR palau  OR tuvalu  OR mauritania  OR mauritius  OR mexico  OR moldova  OR moldovian  OR mongolia  OR montenegro  OR morocco  OR ifni  OR mozambique  OR "portuguese east africa"  OR myanmar  OR burma  OR namibia  OR nepal  OR "netherlands antilles"  OR nicaragua  OR niger  OR nigeria  OR oman  OR muscat  OR pakistan  OR panama  OR "papua new guinea"  OR paraguay  OR peru  OR philippines  OR philipines  OR phillipines  OR phillippines  OR poland  OR "polish people's republic"  OR portugal  OR "portuguese republic"  OR "puerto rico"  OR romania  OR russia  OR "russian federation"  OR ussr  OR "soviet union"  OR "union of soviet socialist republics"  OR rwanda  OR ruanda  OR samoa  OR "pacific islands"  OR polynesia  OR "samoan islands"  OR "navigator island"  OR "navigator islands"  OR "sao tome and principe"  OR "saudi arabia"  OR senegal  OR serbia  OR seychelles  OR "sierra leone"  OR slovakia  OR "slovak republic"  OR slovenia  OR melanesia  OR "solomon island"  OR "solomon islands"  OR "norfolk island"  OR "norfolk islands"  OR somalia  OR "south africa"  OR "south sudan"  OR "sri lanka"  OR ceylon  OR "saint kitts and nevis"  OR "st. kitts and nevis"  OR "saint lucia"  OR "st. lucia"  OR "saint vincent and the grenadines"  OR "saint vincent"  OR "st. vincent"  OR grenadines  OR sudan  OR suriname  OR surinam  OR "dutch guiana"  OR "netherlands guiana"  OR syria  OR "syrian arab republic"  OR tajikistan  OR tadjikistan  OR tadzhikistan  OR tadzhik  OR tanzania  OR tanganyika  OR thailand  OR siam  OR "timor leste"  OR "east timor"  OR togo  OR "togolese republic"  OR tonga  OR "trinidad and tobago"  OR trinidad  OR tobago  OR tunisia  OR turkey  OR turkmenistan  OR turkmen  OR uganda  OR ukraine  OR uruguay  OR uzbekistan  OR uzbek  OR vanuatu  OR "new hebrides"  OR venezuela  OR vietnam  OR "viet nam"  OR "middle east"  OR "west bank"  OR gaza  OR palestine  OR yemen  OR yugoslavia  OR zambia  OR zimbabwe  OR "northern rhodesia"  OR "global south"  OR "africa south of the sahara"  OR "sub saharan africa"  OR "subsaharan africa"  OR "africa, central"  OR "central africa"  OR "africa, northern"  OR "north africa"  OR "northern africa"  OR magreb  OR maghrib  OR sahara  OR "africa, southern"  OR "southern africa"  OR "africa, eastern"  OR "east africa"  OR "eastern africa"  OR "africa, western"  OR "west africa"  OR "western africa"  OR "west indies"  OR "indian ocean islands"  OR caribbean  OR "central america"  OR "latin america"  OR "south and central america"  OR "south america"  OR "asia, central"  OR "central asia"  OR "asia, northern"  OR "north asia"  OR "northern asia"  OR "asia, southeastern"  OR "southeastern asia"  OR "south eastern asia"  OR "southeast asia"  OR "south east asia"  OR "asia, western"  OR "western asia"  OR "europe, eastern"  OR "east europe"  OR "eastern europe"  OR "developing country"  OR "developing countries"  OR "developing nation"  OR "developing nations"  OR "developing population"  OR "developing populations"  OR "developing world"  OR "less developed country"  OR "less developed countries"  OR "less developed nation"  OR "less developed nations"  OR "less developed population"  OR "less developed populations"  OR "less developed world"  OR "lesser developed country"  OR "lesser developed countries"  OR "lesser developed nation"  OR "lesser developed nations"  OR "lesser developed population"  OR "lesser developed populations"  OR "lesser developed world"  OR "under developed country"  OR "under developed countries"  OR "under developed nation"  OR "under developed nations"  OR "under developed population"  OR "under developed populations"  OR "under developed world"  OR "underdeveloped country"  OR "underdeveloped countries"  OR "underdeveloped nation"  OR "underdeveloped nations"  OR "underdeveloped population"  OR "underdeveloped populations"  OR "underdeveloped world"  OR "middle income country"  OR "middle income countries"  OR "middle income nation"  OR "middle income nations"  OR "middle income population"  OR "middle income populations"  OR "low income country"  OR "low income countries"  OR "low income nation"  OR "low income nations"  OR "low income population"  OR "low income populations"  OR "lower income country"  OR "lower income countries"  OR "lower income nation"  OR "lower income nations"  OR "lower income population"  OR "lower income populations"  OR "underserved country"  OR "underserved countries"  OR "underserved nation"  OR "underserved nations"  OR "underserved population"  OR "underserved populations"  OR "underserved world"  OR "under served country"  OR "under served countries"  OR "under served nation"  OR "under served nations"  OR "under served population"  OR "under served populations"  OR "under served world"  OR "deprived country"  OR "deprived countries"  OR "deprived nation"  OR "deprived nations"  OR "deprived population"  OR "deprived populations"  OR "deprived world"  OR "poor country"  OR "poor countries"  OR "poor nation"  OR "poor nations"  OR "poor population"  OR "poor populations"  OR "poor world"  OR "poorer country"  OR "poorer countries"  OR "poorer nation"  OR "poorer nations"  OR "poorer population"  OR "poorer populations"  OR "poorer world"  OR "developing economy"  OR "developing economies"  OR "less developed economy"  OR "less developed economies"  OR "lesser developed economy"  OR "lesser developed economies"  OR "under developed economy"  OR "under developed economies"  OR "underdeveloped economy"  OR "underdeveloped economies"  OR "middle income economy"  OR "middle income economies"  OR "low income economy"  OR "low income economies"  OR "lower income economy"  OR "lower income economies"  OR "low gdp"  OR "low gnp"  OR "low gross domestic"  OR "low gross national"  OR "lower gdp"  OR "lower gnp"  OR "lower gross domestic"  OR "lower gross national"  OR lmic  OR lmics  OR "third world"  OR "lami country"  OR "lami countries"  OR "transitional country"  OR "transitional countries"  OR "emerging economies"  OR "emerging nation"  OR "emerging nations"  OR afghan  OR afghans  OR afghani  OR albanian  OR albanians  OR algerian  OR algerians  OR "american samoan"  OR "american samoans"  OR angolan  OR angolans  OR antiguan  OR antiguans  OR barbudan  OR berbudans  OR argentine  OR argentines  OR argentinian  OR argentinians  OR argentinean  OR argentineans  OR armenian  OR armenians  OR aruban  OR arubans  OR azerbaijani  OR azerbaijanis  OR bahraini  OR bahrainis  OR bangladeshi  OR bangladeshis  OR bangalees  OR bajan  OR bajans  OR belarusian  OR belarusians  OR byelorussian  OR byelorussians  OR belizean  OR belizeans  OR beninese  OR benineses  OR bhutanese  OR bolivian  OR bolivians  OR bosnian  OR bosnians  OR botswana  OR batswana  OR brazilian  OR brazilians  OR brasilian  OR brasilians  OR bulgarian  OR bulgarians  OR burkinabe  OR burkinese  OR burundian  OR burundians  OR "cape verdean"  OR "cape verdeans"  OR "cabo verdean"  OR "cabo verdeans"  OR cambodian  OR cambodians  OR khmer  OR cameroonian  OR cameroonians  OR "central african"  OR "central africans"  OR chadian  OR chadians  OR chilean  OR chileans  OR chinese  OR colombian  OR colombians  OR comorian  OR comorians  OR congolese  OR "costa rican"  OR "costa ricans"  OR ivorian  OR ivorians  OR croatian  OR croatians  OR cuban  OR cubans  OR cypriot  OR cypriots  OR czech  OR czechs  OR djiboutian  OR djiboutians  OR dominican  OR dominicans  OR ecuadorian  OR ecuadorians  OR egyptian  OR egyptians  OR salvadoran  OR salvadorans  OR "equatorial guinean"  OR "equatorial guineans"  OR equatoguinean  OR equatoguineans  OR eritrean  OR eritreans  OR estonian  OR estonians  OR swazi  OR swazis  OR swati  OR swatis  OR ethiopian  OR ethiopians  OR fijian  OR fijians  OR gabonese  OR gabonaise  OR gambian  OR gambians  OR georgian  OR georgians  OR ghanaian  OR ghanaians  OR gibraltarian  OR gibraltarians  OR greek  OR greeks  OR grenadian  OR grenadians  OR guamanian  OR guamanians  OR guatemalan  OR guatemalans  OR guinean  OR guineans  OR "bissau guinean"  OR "bissau guineans"  OR guyanese  OR haitian  OR haitians  OR honduran  OR hondurans  OR hungarian  OR hungarians  OR indian  OR indians  OR indonesian  OR indonesians  OR iranian  OR iranians  OR iraqian  OR iraqians  OR iraqi  OR iraqis  OR manx  OR jamaican  OR jamaicans  OR jordanian  OR jordanians  OR kazakhstani  OR kazakhstanis  OR kenyan  OR kenyans  OR kirabati  OR kirabatian  OR kirabatians  OR "north korean"  OR "north koreans"  OR korean  OR koreans  OR kosovar  OR kosovars  OR kosovan  OR kosovans  OR kyrgyzstani  OR kyrgyzstanis  OR kyrgyz  OR lao  OR laotian  OR laotians  OR latvian  OR latvians  OR lebanese  OR lesothan  OR lesothans  OR lesothonian  OR lesothonians  OR mosotho  OR basotho  OR liberian  OR liberians  OR libyan  OR libyans  OR lithuanian  OR lithuanians  OR macanese  OR macedonian  OR macedonians  OR malagasy  OR madagascan  OR madagascans  OR malawian  OR malawians  OR malaysian  OR malaysians  OR maldivian  OR maldivians  OR malian  OR malians  OR maltese  OR marshallese  OR marshalleses  OR mauritanian  OR mauritanians  OR  mauritian  OR mauritians  OR mexican  OR mexicans  OR micronesian  OR micronesians  OR moldovan  OR moldovans  OR mongolian  OR mongolians  OR mongol  OR montenegrin  OR montenegrins  OR moroccan  OR moroccans  OR mozambican  OR mozambicans  OR burmese  OR myanma  OR namibian  OR namibians  OR nauruan  OR nauruans  OR nepali  OR nepalese  OR "netherlands antillean"  OR "netherlands antilleans"  OR nicaraguan  OR nicaraguans  OR nigerien  OR  nigeriens  OR nigerian  OR nigerians  OR "northern mariana islander"  OR "northern mariana islanders"  OR mariana  OR marianas  OR omani  OR omanis  OR pakistani  OR pakistanis  OR palauan  OR palauans  OR panamanian  OR panamanians  OR "papua new guinean"  OR "papua new guineans"  OR paraguayan  OR paraguayans  OR peruvian  OR peruvians  OR philippine  OR philippines  OR philipine  OR philipines  OR phillipine  OR phillipines  OR phillippine  OR phillippines  OR filipino  OR filipinos  OR filipina  OR filipinas  OR polish  OR pole  OR poles  OR portuguese  OR "puerto rican"  OR "puerto ricans"  OR romanian  OR romanians  OR russian  OR russians  OR "soviet people"  OR "soviet population"  OR rwandan  OR rwandans  OR rwandese  OR ruandan  OR ruandans  OR ruandese  OR samoan  OR samoans  OR "sao tomean"  OR "sao tomeans"  OR santomean  OR santomeans  OR "saudi arabian"  OR "saudi arabians"  OR saudi  OR saudis  OR senegalese  OR serbian  OR serbians  OR montenegrin  OR montenegrins  OR seychellois  OR seychelloise  OR seychelloises  OR "sierra leonean"  OR "sierra leoneans"  OR slovak  OR slovaks  OR slovene  OR slovenes  OR "solomon islander"  OR "solomon islanders"  OR somali  OR somalis  OR "south african"  OR "south africans"  OR "south sudanese"  OR "sri lankan"  OR "sri lankans"  OR ceylonese  OR kittitian  OR kittitians  OR nevisian  OR nevisians  OR "saint lucian"  OR "saint lucians"  OR vincentian  OR vincentians  OR sudanese  OR surinamese  OR surinameses  OR syrian  OR syrians  OR tajik  OR tajiks  OR tajikistani  OR tajikistanis  OR tanzanian  OR tanzanians  OR tanganyikan  OR tanganyikans  OR thai  OR timorese  OR timoreses  OR togolese  OR tongan  OR tongans  OR trinidadian  OR trinidadians  OR tobagonian  OR tobagonians  OR tunisian  OR tunisians  OR turk  OR turks  OR turkish  OR turkmen  OR  turkmens  OR tuvaluan  OR tuvaluans  OR ugandan  OR ugandans  OR ukrainian  OR ukrainians uruguayan  OR uruguayans  OR uzbek  OR uzbeks  OR vanuatu  OR vanuatuan  OR vanuatuans  OR venezuelan  OR venezuelans  OR vietnamese  OR yemeni  OR yemenis  OR yemenite  OR yemenites  OR yemenese  OR yugoslav  OR yugoslavs  OR yugoslavian  OR yugoslavians  OR zambian  OR zambians  OR zimbabwean  OR Zimbabweans)

# **LILACS: 1165 results**

(implementation OR  "Intervention mapping"  OR  "Intervention design"  OR  "Implementation mapping"  OR  "Consolidated framework for implementation research"  OR  "Re-aim"  OR  "Reach effectiveness adoption implementation maintenance"  OR  "ERIC framework"  OR  "Expert recommendations for implementing change" OR  "Theoretical domains framework"  OR  "needs assessment"  OR  "needs assessments"  OR  "program evaluation"  OR  "program sustainability" OR  "Quality Improvement"  OR  "evidence-based practice" ) AND (cancer OR cancers OR oncology OR neoplasm OR neoplasms) AND ( db:("LILACS"))

# **AJOL: 16 results**

(implementation OR  "Intervention mapping"  OR  "Intervention design"  OR  "Implementation mapping"  OR  "Consolidated framework for implementation research"  OR  "Re-aim"  OR  "Reach effectiveness adoption implementation maintenance"  OR  "ERIC framework"  OR  "Expert recommendations for implementing change" OR  "Theoretical domains framework"  OR  "needs assessment"  OR  "needs assessments"  OR  "program evaluation"  OR  "program sustainability" OR  "Quality Improvement"  OR  "evidence-based practice" ) AND (cancer OR cancers OR oncology OR neoplasm OR neoplasms)
