# Supplementary material for: Implementation science frameworks and strategies to promote adoption of and adherence to oncology clinical practice guidelines in low- and middle-income countries: a scoping review
Source: BMC Glob Public Health. 2026 Jul 16;4:67. doi: 10.1186/s44263-026-00297-4 (PMC13378326; doi:10.1186/s44263-026-00297-4)
Supplement: Supplementary file 3 — Supplementary material 3: Grey literature searches [file 44263_2026_297_MOESM3_ESM.docx]

Supplementary material 3: Grey literature searches

**World Health Organization (WHO)**

*(implement* OR guideline* OR program evaluation* OR quality improvement* OR sustainability OR framework* OR needs assessment* OR feasibility OR dissemination) (cancer* OR oncolog*) (low* income countr* OR middle income countr* OR LMIC*) site:www.who.int*

5 results

**National Comprehensive Cancer Network (NCCN)**

*(implement* OR guideline* OR program evaluation* OR quality improvement* OR sustainability OR framework* OR needs assessment* OR feasibility OR dissemination) (cancer* OR oncolog*) (low* income countr* OR LMIC*) site:www.nccn.org*

3 results

**Breast Health Global Initiative (BHGI)**

*(implement* OR guideline* OR program evaluation* OR quality improvement* OR sustainability OR framework* OR needs assessment* OR feasibility OR dissemination) (cancer* OR oncolog*) (low* income countr* OR middle income countr* OR LMIC*) site:www.fredhutch.org*

6 results

**American Society of Clinical Oncology (ASCO)**

*(implement* OR guideline* OR program evaluation* OR quality improvement* OR sustainability OR framework* OR needs assessment* OR feasibility OR dissemination) (cancer* OR oncolog*) (low* income countr* OR middle income countr* OR LMIC*) site:*[*www.asco.org*](http://www.asco.org)

5 results

**American Society of Breast Surgeons (ASBrS)**

*(implement* OR guideline* OR program evaluation* OR quality improvement* OR sustainability OR framework* OR needs assessment* OR feasibility OR dissemination) (cancer* OR oncolog*) (low* income countr* OR middle income countr* OR LMIC*) site:www.breastsurgeons.org*

2 results

**Society of Surgical Oncology (SSO)**

*(implement* OR guideline* OR program evaluation* OR quality improvement* OR sustainability OR framework* OR needs assessment* OR feasibility OR dissemination) (cancer* OR oncolog*) (low* income countr* OR middle income countr* OR LMIC*) site:www.surgonc.org*

1 result

**European Society of Breast Cancer Specialists (EUSOMA)**

*(implement* OR guideline* OR program evaluation* OR quality improvement* OR sustainability OR framework* OR needs assessment* OR feasibility OR dissemination) (cancer* OR oncolog*) (low* income countr* OR middle income countr* OR LMIC*) site:www.eusoma.org*

1 result

**African Organisation of Research and Training in Cancer (AORTIC)**

*(implement* OR guideline* OR program evaluation* OR quality improvement* OR sustainability OR framework* OR needs assessment* OR feasibility OR dissemination) (cancer* OR oncolog*) (low* income countr* OR middle income countr* OR LMIC*) site:aortic-africa.org*

2 results

**Consortium of Universities for Global Health (CUGH)**

*(implement* OR guideline* OR program evaluation* OR quality improvement* OR sustainability OR framework* OR needs assessment* OR feasibility OR dissemination) (cancer* OR oncolog*) (low* income countr* OR middle income countr* OR LMIC*) site:www.cugh.org*

6 results

**Academy Health**

*(implement* OR guideline* OR program evaluation* OR quality improvement* OR sustainability OR framework* OR needs assessment* OR feasibility OR dissemination) (cancer* OR oncolog*) (low* income countr* OR middle income countr* OR LMIC*) site:*[academyhealth.org](http://academyhealth.org)

4 results

**American Public Health Association (global health & implementation science section)**

*(implement* OR guideline* OR program evaluation* OR quality improvement* OR sustainability OR framework* OR needs assessment* OR feasibility OR dissemination) (cancer* OR oncolog*) (low* income countr* OR middle income countr* OR LMIC*) site:www.apha.org*

2 results

**American Society for Radiation Oncology**

*(implement* OR guideline* OR program evaluation* OR quality improvement* OR sustainability OR framework* OR needs assessment* OR feasibility OR dissemination) (cancer* OR oncolog*) (low* income countr* OR middle income countr* OR LMIC*) site:www.astro.org*

1 result

**Cancer Care Ontario**

*(implement* OR guideline* OR program evaluation* OR quality improvement* OR sustainability OR framework* OR needs assessment* OR feasibility OR dissemination) (cancer* OR oncolog*) (low* income countr* OR middle income countr* OR LMIC*) site:www.cancercareontario.ca*

0 results

**European Society of Medical Oncology**

*(implement* OR guideline* OR program evaluation* OR quality improvement* OR sustainability OR framework* OR needs assessment* OR feasibility OR dissemination) (cancer* OR oncolog*) (low* income countr* OR middle income countr* OR LMIC*) site:www.esmo.org*

7 results

**Saint Gallen International Consensus Conference**

*(implement* OR guideline* OR program evaluation* OR quality improvement* OR sustainability OR framework* OR needs assessment* OR feasibility OR dissemination) (cancer* OR oncolog*) (low* income countr* OR middle income countr* OR LMIC*) site:*www.oncoconferences.ch

1 result
